# JAK2-CHK2 signaling safeguards the integrity of the mitotic spindle assembly checkpoint and genome stability

**DOI:** 10.1038/s41419-022-05077-0

**Published:** 2022-07-18

**Authors:** Md Al Nayem Chowdhury, Shih-Wei Wang, Ching-Shu Suen, Ming-Jing Hwang, Yi-An Hsueh, Sheau-Yann Shieh

**Affiliations:** 1 Taiwan International Graduate Program in Molecular Medicine, National Yang Ming Chiao Tung University and Academia Sinica, Taipei, Taiwan; 2 Institute of Biomedical Sciences, Academia Sinica, Taipei, Taiwan

**Keywords:** Checkpoint signalling, Cancer genomics

## Abstract

Checkpoint kinase 2 (CHK2) plays an important role in safeguarding the mitotic progression, specifically the spindle assembly, though the mechanism of regulation remains poorly understood. Here, we identified a novel mitotic phosphorylation site on CHK2 Tyr156, and its responsible kinase JAK2. Expression of a phospho-deficient mutant CHK2 Y156F or treatment with JAK2 inhibitor IV compromised mitotic spindle assembly, leading to genome instability. In contrast, a phospho-mimicking mutant CHK2 Y156E restored mitotic normalcy in JAK2-inhibited cells. Mechanistically, we show that this phosphorylation is required for CHK2 interaction with and phosphorylation of the spindle assembly checkpoint (SAC) kinase Mps1, and failure of which results in impaired Mps1 kinetochore localization and defective SAC. Concordantly, analysis of clinical cancer datasets revealed that deletion of *JAK2* is associated with increased genome alteration; and alteration in *CHEK2* and *JAK2* is linked to preferential deletion or amplification of cancer-related genes. Thus, our findings not only reveal a novel JAK2-CHK2 signaling axis that maintains genome integrity through SAC but also highlight the potential impact on genomic stability with clinical JAK2 inhibition.

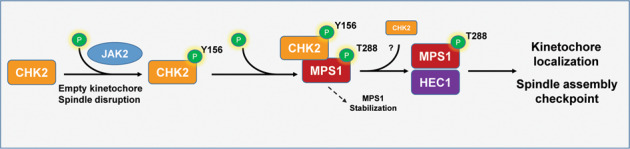

## Introduction

Enzymatic regulation of kinases and substrates is the key component of various cellular signaling pathways, including those involved in tumorigenesis and cancer. To maintain genomic stability, cells follow a well-controlled cell cycle and a tightly regulated cell scanning process [1, 2]. Abnormal surveillance, originating primarily due to genetic mutation, is considered to be the underlying cause of human cancers. Understanding these checkpoints is not only essential for disease management but could also have a significant impact on the development of treatment strategies [3].

The Ser/Thr kinase CHK2 is a well-known arbitrator of diverse cellular genotoxic stresses [4, 5]. Upon DNA damage, CHK2 is phosphorylated and activated by Ataxia-telangiectasia-mutated (ATM) and ataxia telangiectasia and Rad3-related (ATR) proteins in a cell cycle and damage-specific manner. The N-terminal SCD (SQ/TQ cluster) and FHA (Forkhead-associated) domains and the C-terminal catalytic domain make CHK2 a versatile substrate and kinase [4–6]. In response to ionizing radiation (IR), ATM phosphorylates CHK2 on T68 residue in the SCD domain, which mediates dimerization and autophosphorylation required for full activation of CHK2 [7, 8]. CDC25A, p53, BRCA1, E2F1, Mps1, and PARP1, to name a few, are known CHK2 substrates involved in the regulation of cell cycle, damage response, DNA repair, and apoptosis-related signaling [9–17]. Mutations in CHK2 have been discovered in human cancers and cancer predisposition syndromes [4, 18].

Studies conducted by our group as well as others have resulted in a considerable understanding of CHK2-mediated regulation. For instance, CHK2-mediated p53 C-terminal phosphorylation regulates the acetylation and activity of p53 [12]. Through the phosphorylation of BRCA1 and XRCC1, CHK2 participates in the precise end-joining of DNA double-strand breaks [19] and base excision repair [20], respectively. More recently, we have also observed that CHK2-mediated PARP1 phosphorylation in oxidative stress controls PARP1 cellular localization and activity [17]. Regarding chromosome stability, Stolz et al. have shown the association of CHK2 deficiency with abnormal spindle assembly and chromosome missegregation and identified BRCA1 as a probable mitotic target of CHK2, independent of DNA damage [21]. In addition, our group demonstrated that the phosphorylation of the spindle checkpoint kinase Mps1 by CHK2 on Thr288 not only stabilizes Mps1 but also promotes its interaction with HEC1 and in turn, its kinetochore localization in mitosis, indicating that Mps1 can also be a mitotic target of CHK2 [15, 16]. However, it remains unclear how CHK2 is activated in mitosis in the absence of DNA damage.

CHK2 is phosphorylated at Ser or Thr residues for different regulatory pathways. Until now, the evidence of tyrosine phosphorylation in CHK2 has been scarce. Previously, Y220 [22] and Y390 [23] sites were detected by mass spectrometry analysis. Y220 appears to be an autophosphorylation site, and its function remains to be determined. Y390 resides in the T-loop, and its phosphorylation is reduced by IR [23]. Notably, the Y390F mutation abolished the kinase activity, suggesting that the residue and not necessarily its phosphorylation is involved in the kinase activity of CHK2.

To address how CHK2 is regulated in mitosis independently of DNA damage, we posited that a yet to be identified kinase could be acting upstream of CHK2 during mitosis. To search for such a kinase, we employed a bottom-up approach by first determining if any M phase-specific CHK2 phosphorylation exists. Consequently, we discovered a previously unreported CHK2 tyrosine phosphorylation site, Y156, and further explored the would-be kinase for this site. We characterized the functional roles of Y156 phosphorylation in mitosis. We observed an essential role of phosphorylation involving this site as well as its kinase JAK2 in maintaining the integrity of the spindle assembly checkpoint.

## Results

### CHK2 is phosphorylated at tyrosine 156 in nocodazole-arrested M phase cells

To determine whether there is any M phase-specific CHK2 phosphorylation, ectopically expressed CHK2 was immunoprecipitated from control or nocodazole (Noc)-treated HEK293T cells and subjected to mass spectrometry analysis. As a result, numerous phosphorylated residues were detected, mostly serine and threonine (Fig. S1A). Notably, two phosphorylated tyrosine residues, Tyr156 (Y156) and Tyr159 (Y159) were detected, with Y156 being specific to the nocodazole-treated sample (Fig. S1B). Sequence alignment showed that Y156 was conserved among various species (Fig. S1C).

**Fig. 1:**
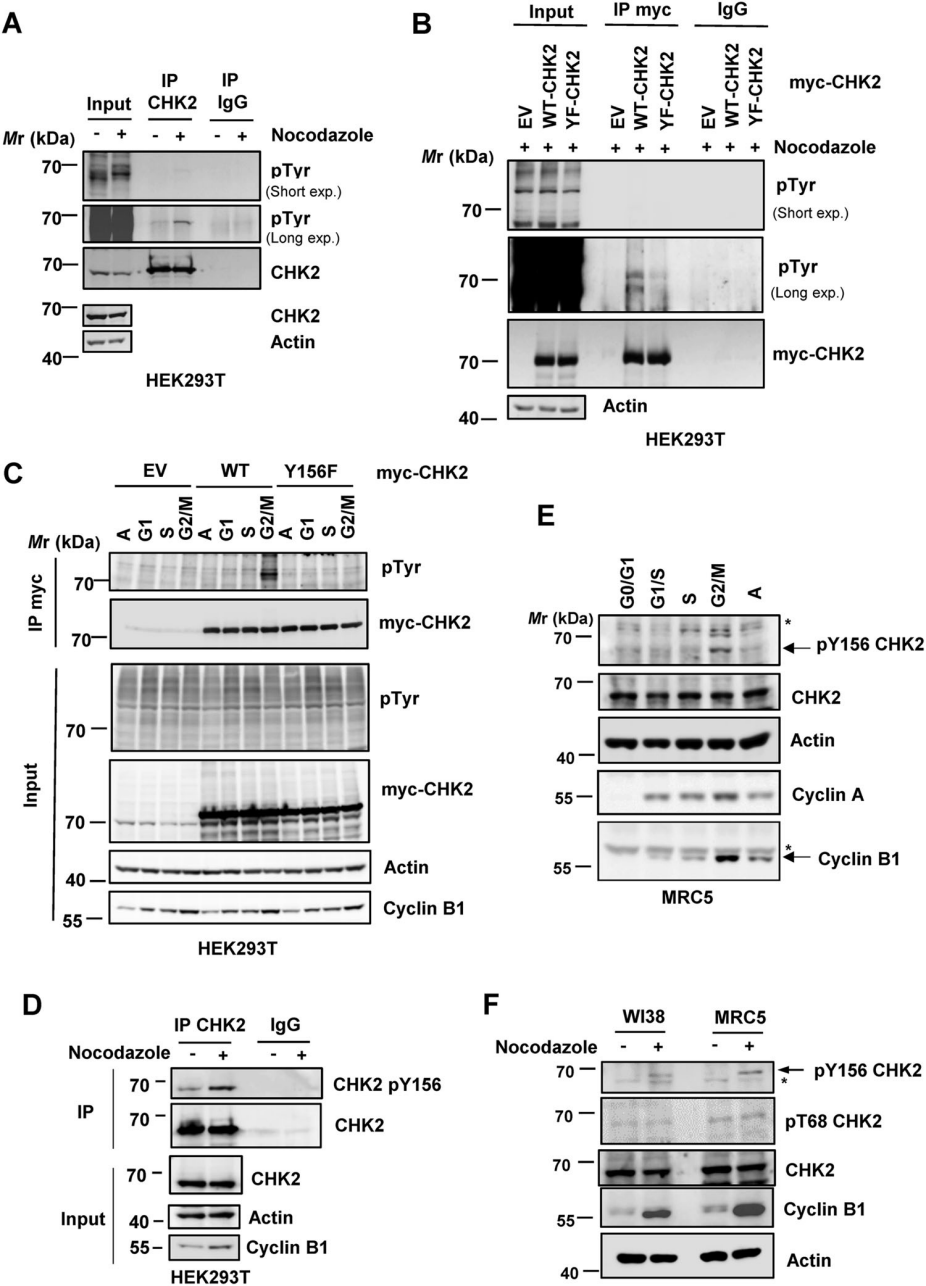
CHK2 is phosphorylated at Tyr156 during mitosis. **A** Endogenous CHK2 is tyrosine-phosphorylated upon nocodazole treatment. HEK293T cells were treated with nocodazole (50 ng/ml) for 16 h, and lysates were prepared for immunoprecipitation and western blotting using the indicated antibodies. **B**, **C** Tyrosine phosphorylation in CHK2 in nocodazole-arrested cells (**B**) or G2/M phase cells (**C**) was diminished by the Y156F mutation. HEK293T cells were transfected with either myc-tagged WT or Y156F CHK2, and lysates were subjected to immunoprecipitation using the anti-myc antibody. Cells in different phases of the cell cycle in (**C**) were collected using double thymidine block and release. **D** Phosphorylation of endogenous CHK2 at Y156 was increased by nocodazole treatment in HEK293T. **E** CHK2 Y156 is phosphorylated in the G2/M normal human fibroblasts MRC5. Cells were synchronized by serum starvation. **F** Phosphorylation of endogenous CHK2 at Y156 was increased by nocodazole treatment in normal human fibroblasts WI38 and MRC5. For the detection of tyrosine phosphorylation, cells were treated with 1 mM pervanadate for 10 min prior to collection. EV, empty vector. “*” indicates a non-specific band.

To further explore this relatively rare tyrosine phosphorylation event, immunoblot analysis was conducted with a phospho-Tyr (pY)-specific antibody. The results showed that endogenous CHK2 is Tyr-phosphorylated, which can be further enhanced by Noc treatment (Fig. 1A). This phosphorylation was markedly reduced when Y156 was mutated to non-phosphorylatable phenylalanine (Y156F) (Fig. 1B), indicating that Y156 may be a genuine phosphorylation site in Noc-arrested cells. Moreover, the Y156F mutation also diminished M phase Tyr phosphorylation following release from the double thymidine block (Fig. 1C). Note that Tyr phosphorylation in G1 and S cells was not affected by Y156F mutation (Fig. 1C), suggesting that CHK2 was also phosphorylated on other Tyr residues, and pY156 appeared to be an M phase-specific event.

To further validate pY156, a pY156-specific antibody was generated, of which the specificity was verified by dot blot (Fig. S2A). Importantly, the antibody preferentially recognized endogenous CHK2 from Noc-treated cells compared to the untreated control (Fig. S2B and Fig. 1D). Collectively, these data suggest that CHK2 Y156 phosphorylation can be an M phase-specific event. Importantly, in normal human fibroblasts MRC5 and WI38, pY156 was also detected in the unperturbed G2/M phase as well as in the Noc-arrested stage (Fig. 1E, F), thus excluding this is a cancer cell-specific event.

### CHK2 Y156 phosphorylation is required for proper mitotic progression

**Fig. 2:**
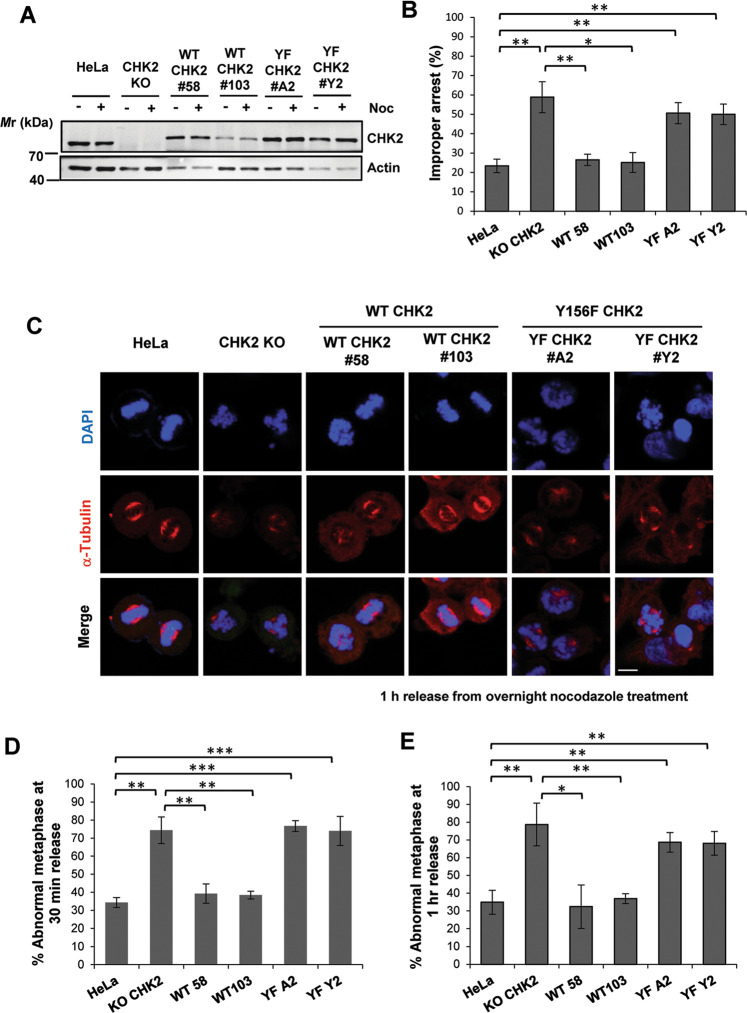
Proper chromosome alignment requires CHK2 Y156 phosphorylation. **A** Western blots showing CHK2 expression in parental HeLa, HeLa with CHK2 knockout (KO), and KO cells reexpressing WT (clones #58, #103) or Y156F CHK2 (clones #A2, Y2). **B** Proportions of cells that were not properly arrested by overnight nocodazole treatment (25 ng/mL). Representative images for improper arrest are shown in Fig. S3B. Shown is mean ± SD from four independent experiments with each 50–60 cells analyzed. **C**–**E** Chromosome alignment after release from nocodazole-mediated mitotic arrest as assessed using confocal microscopy. Representative images captured after 1 h following the release are shown in (**C**). Increased chromosome misalignment was observed in KO cells at 30 min (**D**) and 1 h (**E**) after release, which can be rescued by the reexpression of WT but not Y156F CHK2. Representative images for improper chromosome alignment are shown in Fig. S3C. Mean ± SD from four independent experiments is shown with each 50–60 cells examined. Scale bar, 10 μM.

The depletion of CHK2 has been shown to cause delayed mitotic progression, abnormal chromosome alignment, and defective spindle assembly checkpoint (SAC) in HCT116 cells [21]. To determine whether Y156 phosphorylation plays any role in this, we reexpressed either WT or Y156F CHK2 in CHK2 knockout (KO) HeLa cells that we previously constructed [17] (Fig. 2A). Cells were treated with Noc (25 ng/mL) overnight to arrest them in prometaphase, followed by a release for 30 min or 1 h (Fig. S3A). Consistent with earlier reports [16, 21, 24], more than 50% of abnormal mitotic arrest was observed in CHK2 KO cells, which was completely corrected in CHK2 WT cells but not in CHK2 Y156F cells (Fig. 2B and Fig. S3B). For those that did properly arrest, over 70% were in abnormal metaphase (viz mono- and multipolar spindle, misaligned chromosome; Fig. S3C) upon release for 30 min or 1 h in CHK2 KO cells compared to <40% in parental HeLa. This abnormality was largely corrected in CHK2 WT but not in Y156F cells (Fig. 2C–E), suggesting the importance of Y156 phosphorylation in safeguarding the assembly of mitotic spindles.

### Bioinformatics approach for the identification of tyrosine kinases that interact with CHK2

To identify tyrosine kinases that may phosphorylate CHK2 Y156, bioinformatics approach was taken (Fig. S4A). Our strategy was to first find those coexpressed with CHK2 in the three cancer cell lines, namely, HeLa, HEK293, and HCT116. Analysis was conducted using the microarray gene expression data of the three cancer cell lines downloaded from the NCBI gene expression omnibus (GEO, https://www.ncbi.nlm.nih.gov/geo/) [25] and the RNA-seq data of five similar cancer cell lines downloaded from DepMap portal (https://depmap.org/portal/) [26] (Table S1). The 26 tyrosine kinases listed on the KinasePhos2.0 (http://kinasephos2.mbc.nctu.edu.tw/) [27] were used to search for their genes and gene probes in both RNA-seq and microarray datasets. The resulting 41 tyrosine kinase genes were then subjected to gene co-expression analysis by computing the pairwise Spearman’s rank correlation between *CHK2* and tyrosine kinase genes using the Hmisc and corrplot package (https://github.com/taiyun/corrplot) in R software [28, 29] (Fig. S4B).

Five genes (*EPHA1*, *RET*, *FES*, *JAK2*, and *WEE1*) from the RNA-seq datasets and 19 genes from the microarray datasets showed a strong positive correlation for coexpression with *CHK2* with a Spearman’s correlation value > 0.7 and an associated *P*-value < 0.05 (Fig. S4B and Table S2). Furthermore, since checkpoint-mediated cell cycle arrest and phosphorylation of CHK2 Y156 occur in the nucleus, annotations of subcellular locations for these tyrosine kinases were extracted from UniProt (https://www.uniprot.org/) [30] to narrow our candidates. Of them, we selected JAK2 for further examination. WEE1, the only tyrosine kinase found in both microarray and RNA-seq datasets, is involved in pathways of checkpoint regulation [31, 32], which, to some extent, serves to validate our bioinformatics approach.

### JAK2 phosphorylates CHK2 at Y156

**Fig. 3:**
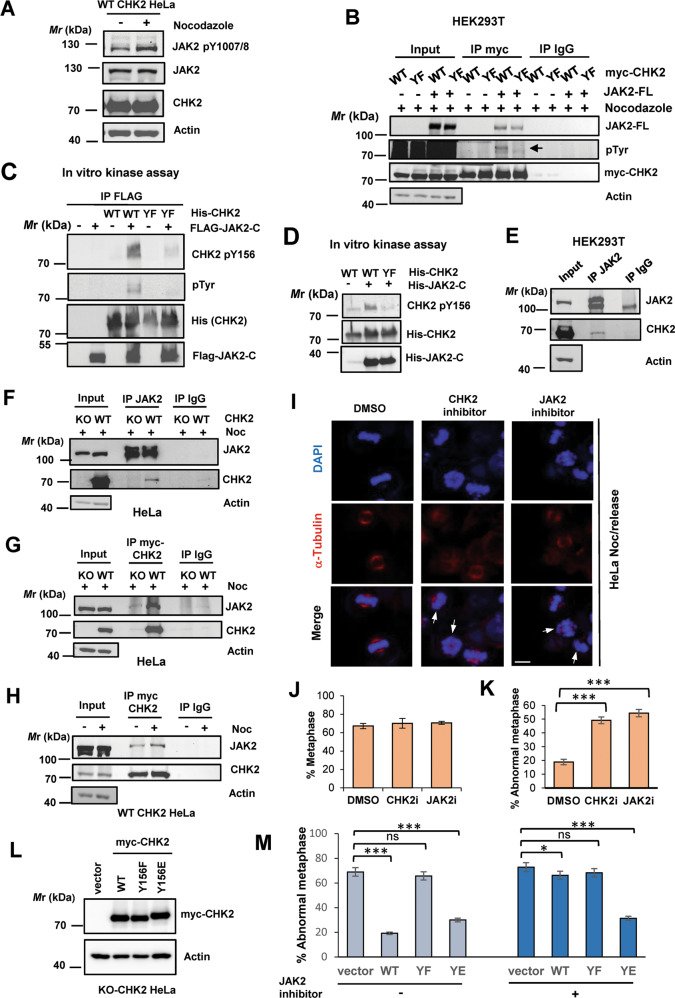
JAK2 phosphorylates CHK2 at Y156 to maintain the spindle assembly checkpoint. **A** JAK2 activity was increased by nocodazole treatment, as evidenced by enhanced JAK2 Y1007/8 phosphorylation (pY1007/8). CHK2 KO cells stably re-expressing myc-tagged WT CHK2 were treated with nocodazole overnight. Lysates were prepared for western blotting using the indicated antibodies. **B** Co-expressed full-length JAK2 (JAK2-FL) promoted tyrosine phosphorylation in WT but not Y156F CHK2 in HEK293T cells. **C**, **D** In vitro kinase assays showing that immunoprecipitated FLAG-JAK2-C (amino acids 747–1132) (**C**) or recombinant His-JAK2-C (amino acids 843–1122) phosphorylated recombinant His-CHK2 at Y156. **E** Interaction of endogenous JAK2 with CHK2 in HEK293T cells as assessed by co-immunoprecipitation. Cells were treated with nocodazole overnight. **F**, **G** Co-immunoprecipitation of JAK2 and CHK2 from HeLa CHK2 KO cells stably expressing myc-CHK2 WT with anti-JAK2 (**F**) or anti-myc (**G**) antibody. Cells were treated with nocodazole overnight. (H) Nocodazole treatment can moderately increase JAK2-CHK2 interaction. Lysates as described in (**A**) were used for immunoprecipitation. **I**–K Inhibition of JAK2 and CHK2 led to abnormal metaphase. Nocodazole-arrested cells were released into the medium containing either CHK2 inhibitor II (5 μM) or JAK2 inhibitor IV (2.5 μM) for 1 h. Representative images (**I**) were captured after 1 h following release. White arrows indicate misaligned chromosomes. Percent cells in metaphase were determined and shown in (**J**), of which those with abnormal metaphase were counted and shown in (**K**). Scale bar, 10 μM. **L**, **M** The phospho-mimicking CHK2 Y156E mutant restored normal metaphase even in the presence of JAK2 inhibitor. Wild type or mutant CHK2 was transiently expressed in CHK2 KO cells, followed by overnight Noc treatment. Images were taken 1 h following release in the presence or absence of JAK2 inhibitor IV. mCherry was coexpressed to mark the transfected cells.

Among the candidate tyrosine kinases predicted using the bioinformatics approach, JAK2 caught our attention as the loss of JAK2 has been reported to cause mitotic errors [33]; and active JAK2, similar to active CHK2, localizes to centrosome [33–35]. Indeed, JAK2 activity was increased by Noc treatment, as evidenced by increased Y1007/8 phosphorylation (Fig. 3A), and full-length JAK2 increased Tyr phosphorylation of coexpressed WT CHK2 but not the Y156F mutant though the interaction remained unaffected (Fig. 3B). Conversely, knockdown of JAK2 by RNA interference reduced CHK2 pY156 after Noc treatment (Fig. S2C). In in vitro kinase assays, immunoprecipitated JAK2 C-terminal kinase domain (amino acids 747-1132) [36] phosphorylated recombinant CHK2 at Y156 but did not phosphorylate the Y156F mutant (Fig. 3C). Importantly, this activity could be inhibited by a JAK2-specific inhibitor (Fig. S2D and S2E). Moreover, a recombinant His-tagged JAK2 C-terminal kinase domain (amino acids 843–1122) also phosphorylated WT CHK2 at Y156, although to a lesser degree (Fig. 3D). Taken together, these data suggest that JAK2 can directly phosphorylate CHK2 at Y156.

In addition to JAK2, we also tested WEE1, SRC, and EGFR. WEE1 did not phosphorylate the coexpressed WT or Y156F CHK2 upon Noc treatment (Fig. S5A). Recombinant SRC did not phosphorylate CHK2 in vitro, although it was capable of autophosphorylation (Fig. S5B). Furthermore, EGF stimulation promoted AKT phosphorylation but had no apparent effect on CHK2 tyrosine phosphorylation (Fig. S5C), suggesting that EGFR may not be the CHK2 kinase.

We also investigated whether JAK2 interacts with CHK2. Both WT and Y156F CHK2 interacted with the coexpressed full-length JAK2 as demonstrated with the coimmunoprecipitation assay (Fig. 3B). Endogenous JAK2 and CHK2 could also be coimmunoprecipitated, as assessed with 293T cells or HeLa CHK2 WT cells (Fig. 3E–G), independent of Noc (Fig. 3H) and JAK2 inhibitor (Fig. S2F) treatment. These data suggest that interaction of the two proteins could occur without phosphorylation.

### JAK2-mediated CHK2 Y156 phosphorylation is required for proper chromosome alignment in metaphase

Next, we compared the mitotic progression of JAK2-inhibited or CHK2-inhibited cells. HeLa cells arrested overnight with Noc were released into a medium containing either CHK2 inhibitor II (5 μM) or JAK2 inhibitor IV (2.5 μM) for 1 h. The results showed that even though all cells entered metaphase similarly, a significantly higher proportion of abnormality was observed in cells with CHK2 or JAK2 inhibition (Fig. 3I–K). Furthermore, a phospho-mimicking CHK2 mutant Y156E restored the normal metaphase, even in the presence of the JAK2 inhibitor (Fig. 3L, M). Cumulatively, these results support the hypothesis that JAK2 and CHK2 act in the same pathway ensuring proper mitotic progression, most likely through the phosphorylation of CHK2 Y156.

### JAK2 and CHK2 colocalize at the centrosome during mitosis

**Fig. 4:**
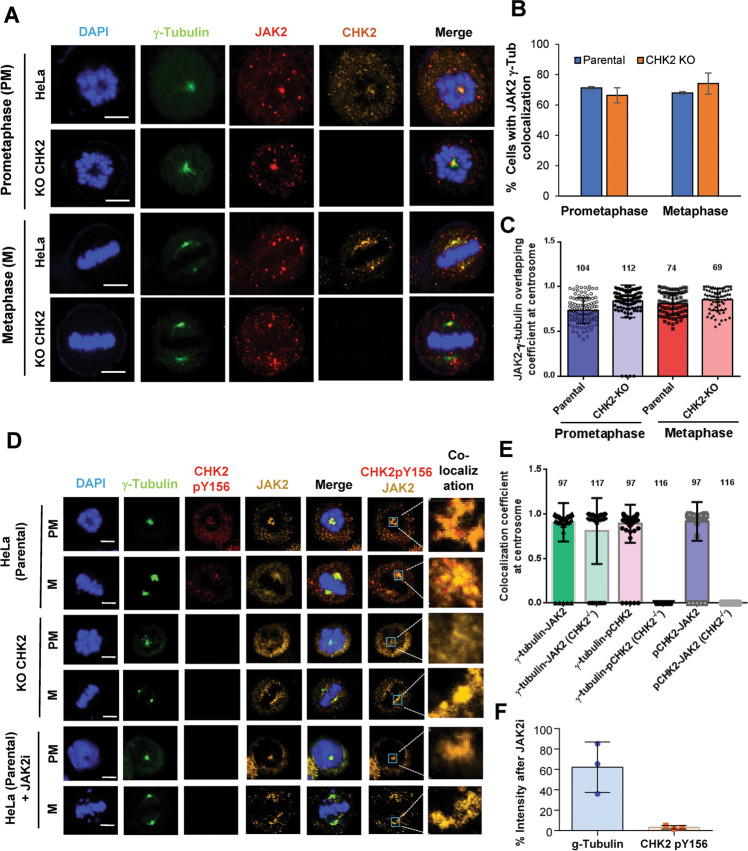
Colocalization of JAK2 with CHK2 during mitosis. **A**–**C** Costaining of JAK2 and γ-tubulin at the centrosome in mitotic cells. Parental or CHK2 KO HeLa cells were treated with nocodazole overnight, without release (prometaphase) or released for 1 h (metaphase), and costained with anti-γ-tubulin (a centrosome marker), anti-JAK2, and anti-CHK2 antibodies (**A**). Proportion of cells with JAK2 localized at the centrosome was calculated and their overlapping coefficients with γ-tubulin were measured and shown in (**B**), (**C**), respectively. Data were collected from three independent experiments, each of 40–50 cells. **D**, **E** Costaining of JAK2 and CHK2 pY156 at centrosomes. Confocal microscopy was done as described above except that the CHK2 pY156 antibody was used (**D**). Overlapping coefficients between JAK2, γ-tubulin, and CHK2 pY156 staining are analyzed using Zen Black 3.0 SR and shown in (**E**). **F** CHK2 pY156 staining was significantly reduced in cells treated with the JAK2 inhibitor. Averaged centrosomal staining intensity from 17 to 20 cells is shown as a percent of untreated control. Mean ± SD from three independent experiments are displayed. Numbers in the scatter plots (**C**), (**E**) represent cell counts. Scale bar, 5 μM.

Upon examination of their cellular localization, CHK2 and JAK2 were both detected at the centrosome during mitosis in HeLa cells, though both also showed additional residence in cells. Image analysis showed a high overlapping coefficient between JAK2 and the centrosomal marker γ-tubulin (Fig. 4A, B), which appeared to be independent of CHK2 (Fig. 4B, C). Significantly, JAK2 costained with CHK2 pY156 at the centrosome during mitosis (Fig. 4D, E), and staining with CHK2 pY156 was markedly reduced in cells treated with the JAK2 inhibitor (Fig. 4F). Similar colocalization events were also observed in the normal human fibroblasts MRC5 with CHK2 knockdown as a negative control (Fig. S6). These data demonstrated the close proximity of these two proteins during mitosis that would allow phosphorylation of CHK2 Y156 by JAK2.

### Mps1-CHK2 interaction and Mps1 kinetochore localization are impaired in the absence of Y156 phosphorylation

**Fig. 5:**
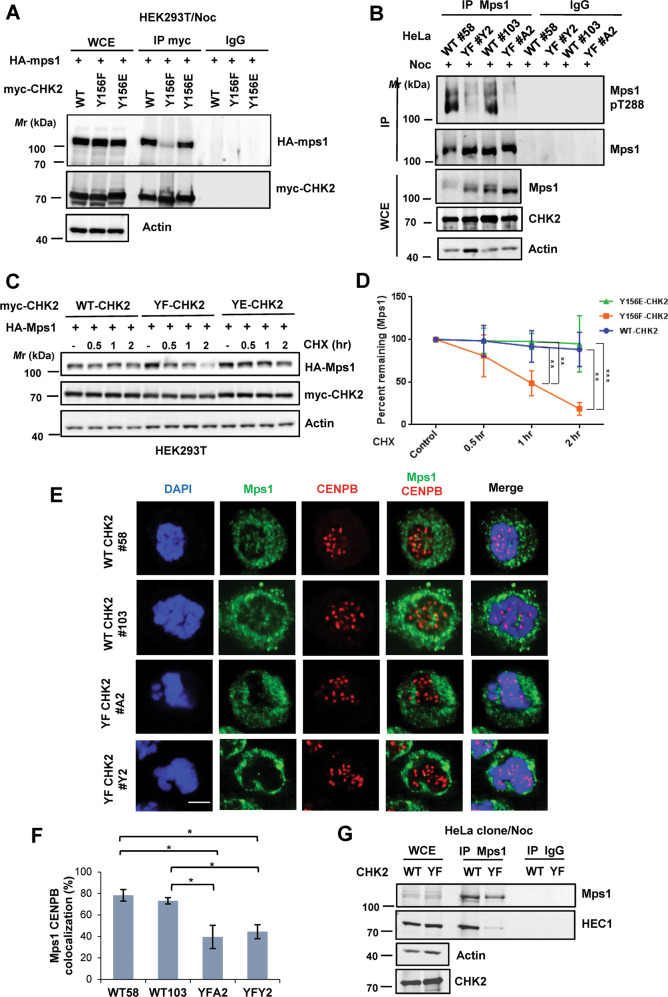
CHK2 Y156 phosphorylation promotes Mps1 T288 phosphorylation and kinetochore localization. **A** Interaction of CHK2 Y156F with Mps1 was reduced as compared to that observed with CHK2 WT and CHK2 Y156E. Co-transfection and co-IP were performed with HEK293T cells. Cells were treated with 50 ng/ml nocodazole overnight before collection. **B** Nocodazole-induced Mps1 T288 phosphorylation was decreased in CHK2 Y156F HeLa cells. Cells were treated with nocodazole overnight. **C**, **D** Mps1 stability was lower with Y156F CHK2 than with WT and Y156E CHK2 coexpression in HEK293T cells. Cells were treated with 50 μg/ml cycloheximide (CHX) and collected at the indicated time points (**C**). Mps1 bands were quantified and normalized to those of Actin. Mean ± SD from four independent experiments is shown in (**D**). **E**, **F** Kinetochore localization of Mps1 was impaired in CHK2 Y156F HeLa cells. CHK2 WT or Y156F HeLa cells were treated with nocodazole overnight and processed for confocal microscopy by co-staining with anti-Mps1 and anti-CENPB (a kinetochore marker) antibodies. Representative images are shown in (**E**). Scale bar, 5 μM. Percent cells with more than five colocalized kinetochores were determined and are shown in (**F**). **G** Interaction between HEC1 and Mps1 was comparatively higher in the stable cell line with WT CHK2 (#103) than that observed in Y156F CHK2 (#A2). Cells were treated with nocodazole overnight.

Previously, we and others have shown that CHK2 stabilizes the spindle assembly checkpoint kinase Mps1, and CHK2-mediated Mps1 T288 phosphorylation is required for Mps1 kinetochore localization in the event of mitotic disruption [15, 16, 24]. In light of the mitotic defects observed in CHK2 Y156F HeLa cells, we sought to compare WT, the phospho-deficient Y156F, and the phospho-mimicking Y156E CHK2 with respect to their interactions with Mps1. The results from coexpression and coimmunoprecipitation showed that the interaction was weakened with Y156F mutation in CHK2 which was restored with the Y156E mutation (Fig. 5A). Consistently, Mps1 T288 phosphorylation was reduced in Noc-treated CHK2 Y156F HeLa cells (Fig. 5B), even though WT and Y156F CHK2 were equally active in substrate phosphorylation (Fig. S7A) and autophosphorylation in vitro (Fig. S7B, S7C). Besides, the stability of Mps1 was significantly reduced when coexpressed with Y156F CHK2 compared with that with WT and the Y156E mutant (Fig. 5C, D). Consequently, kinetochore localization of Mps1 was significantly dampened in the two Y156F clones compared to that observed in the two WT clones (Fig. 5E, F, and Fig. S8), consistent with the reduced interaction between HEC1 and Mps1 in CHK2 Y156F cells (Fig. 5G). Together, these results provide a mechanistic explanation for the mal-regulated spindle checkpoint observed in CHK2 Y156F phosphorylation-deficient cells, illustrating the importance of Y156 phosphorylation in these events.

Unlike the earlier studies by Stolze et al. [21], we did not find CHK2 T68 phosphorylation (pT68) to be involved in mitotic regulation. In our experiments, pT68 was not induced by Noc in HeLa (Fig. S9A) or in the normal human fibroblasts WI38 and MRC5 (Fig. 1F). CHK2 T68A can be similarly phosphorylated by JAK2 on Y156 (Fig. S9B), was capable of interacting with and phosphorylating Mps1 (Fig. S9C and S9D), and did not appear to interfere with Mps1-HEC1 interaction like the Y156F mutant does (Fig. S9E). Furthermore, the stability of Mps1 was similar when coexpressed with either WT or T68A CHK2 (Fig. S9F and S9G). Our data suggest that pY156 and pT68 of CHK2 are independently regulated, with pY156 playing a predominant role in regulating Mps1 kinetochore localization.

### Accelerated mitotic exit and increased micronuclei in KO-CHK2 and Y156F CHK2 HeLa cells

**Fig. 6:**
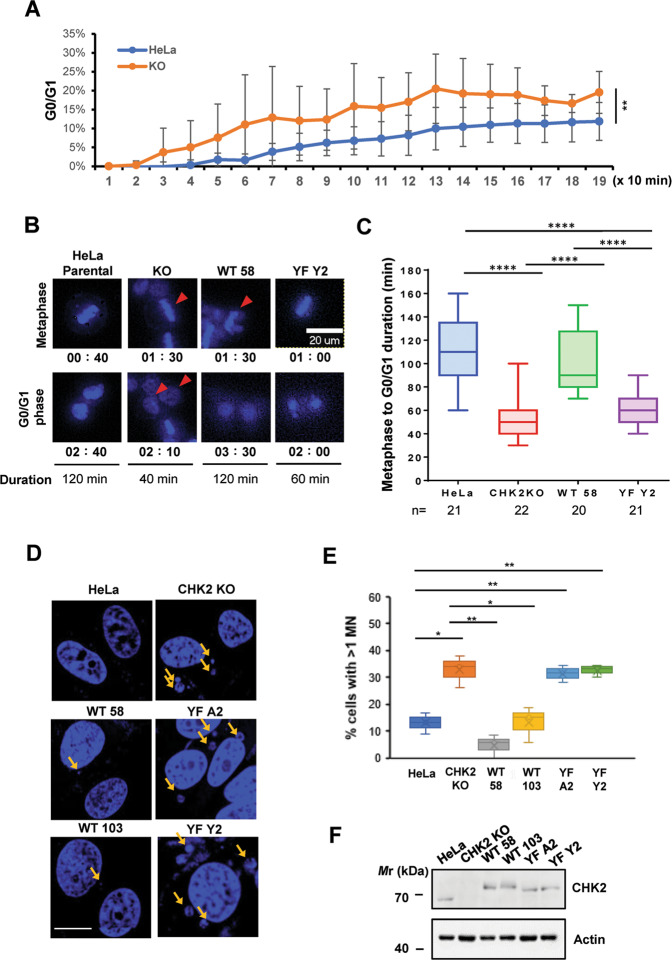
Accelerated mitotic exit coupled with genome instability in CHK2 KO and CHK2 Y156F HeLa cells. **A**–**C** An accelerated mitotic exit was observed in CHK2 KO and CHK2 Y156F (#Y2) cells compared to parental HeLa and CHK2 WT (#58) cells. Cells were released from overnight nocodazole treatment, stained with Hoechst 33342, and subjected to high-content live-cell imaging. A comparison of progression toward G0/G1 between parental and CHK2 KO, analyzed using MetaXpress, is shown in (**A**) as mean ± SD from three independent experiments. Representative division with the time spanning between metaphase and cytokinesis is shown (**B**). Results of the analysis of 20–22 cells are shown in (**C**). Arrows indicate those cells that have been traced. Scale bar, 20 μM. **D**–**F** Enhanced genome instability was demonstrated by increased micronuclei in CHK2 KO and CHK2 Y156F HeLa cells. Cells were assessed for the presence of micronuclei two days after release from nocodazole-mediated arrest. DAPI-stained representative images are shown in (**D**) with arrows indicating micronuclei. Scale bar, 10 μM. Cells with more than one micronucleus were counted, and results from three independent experiments are shown in (**E**). Expression of CHK2, as assessed by western blot, is demonstrated in (**F**).

Given the defects observed in CHK2 KO and CHK2 Y156F cells, we sought to compare the duration of mitosis between metaphase and cytokinesis in these cells, with the premise that the impaired mitotic checkpoint may allow cells to exit mitosis earlier. Cells were released from nocodazole, stained briefly with Hoechst, and subjected to time-lapse live imaging with images captured every 10 min for 3 to 4 h (Fig. 6A–C). Interestingly, the CHK2 KO cells excelled from the start (Fig. 6A) and exited mitosis almost twice as fast as the parental HeLa cells (Fig. 6B, C, and supplemental movies), suggesting that CHK2 was required for timing and safeguarding the mitotic progression. Importantly, reexpression of WT CHK2 but not Y156F CHK2 restored the rate to that observed in parental HeLa cells (Fig. 6C). This observation supports the requirement of Y156 phosphorylation in the regulation of mitotic progression.

The accelerated mitotic exit and abnormal chromosome alignment in KO and Y156F cells led us to speculate that genome integrity may be subverted. The formation of micronuclei (MN), a result of chromosome misalignment and ill-fated segregation, is a sign of genome instability [37, 38]. Therefore, we compared the genome features of parental HeLa, CHK2 KO cells, and KO cells reexpressing WT or Y156F CHK2 two days after release from prolonged (overnight) nocodazole treatment. As expected, CHK2 KO cells demonstrated a higher MN formation compared to the parental HeLa cells. This could be rescued by reexpressing WT but not Y156F CHK2 (Fig. 6D, E), even though comparable amounts of proteins were expressed (Fig. 6F). Taken together, these results demonstrate that JAK2-mediated CHK2 Y156 phosphorylation is required for the mitotic checkpoint and keeping the genome intact.

### Increased genome alterations in human cancers with altered *JAK2* and *CHEK2*

**Fig. 7:**
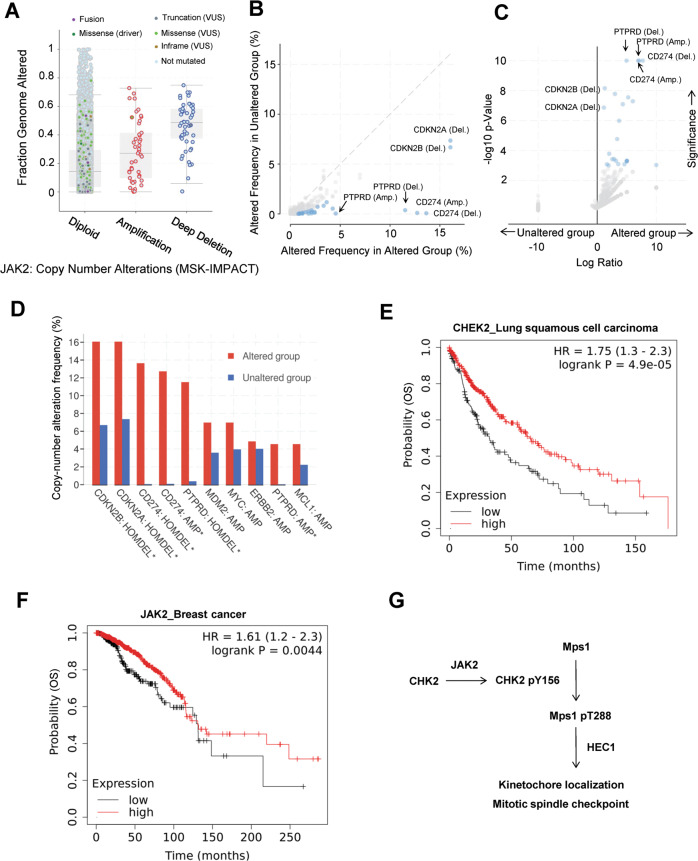
Genome alterations in cancer patient specimens with altered *CHEK2* and *JAK2*. **A** Deletion of *JAK2* is associated with increased genome alterations. Datasets were analyzed using the cBioportal for Cancer Genomics platform (https://www.cbioportal.org). *n* = 10939. **B**–**D** Copy number alterations of genes in relation to mutations and copy number alteration of *CHEK2* and *JAK2*. The gene alteration frequency in groups with altered *CHEK2* and *JAK2* vs. unaltered groups was compared and shown in scatter plot (**B**) and volcano plots (**C**). Genes with the highest frequency were selected and compared between the two groups in (**D**). Datasets from seven pan-cancer studies were analyzed. Altered group, *n* = 703. Unaltered group, *n* = 37045. **E**, **F** Higher expression of *CHEK2* and *JAK2* correlates with better survival among patients with lung squamous cell carcinoma (**E**) and breast cancer (**F**), respectively. Overall survival among patients was analyzed using Kaplan–Meier Plotter (http://kmplot.com/analysis) with pan-cancer RNA-seq datasets. Shown are the results from the analysis of lung squamous cell carcinoma (*n* = 501) and breast cancer (*n* = 1090) for the expression of *CHEK2* (**E**) and *JAK2* (**F**), respectively. G Summary of the JAK2-CHK2-Mps1 signaling axis in the regulation of spindle checkpoint.

To determine the clinical implications of our findings, we analyzed publicly available cancer patient datasets [39–45] using the cBioportal for Cancer Genomic platform (https://www.cbioportal.org). In line with the observations of HeLa cells, deletion of JAK2 in human cancers was associated with increased genome alterations (pan-cancer, *n* = 10939) (Fig. 7A), and alterations of *JAK2*, including deep deletion, amplification, and mutations, were found in a spectrum of human metastatic cancers (Fig. S10A). Furthermore, upon comparing the groups demonstrating alterations of *CHEK2* and *JAK2* (mutations and copy number variation, *n* = 703) with an unaltered group (*n* = 37045), several cancer-related genes were found to be preferentially altered in the former (Fig. 7B–D). Significantly, the deletion of *CDKN2A*/p16 and *CDKN2B*/p15 and deletion and amplification of *CD274*/PD-L1 and *PTPRD* were preferentially elevated in the group with altered *CHEK2* and *JAK2* (Fig. 7D). Consequently, higher *CHEK2* and *JAK2* expression correlated with better survival among patients with lung squamous cell carcinoma (Fig. 7E), breast cancer (Fig. 7F), rectal adenocarcinoma (Fig. S10B and S10C), ovarian cancer (Fig. S10D and S10E), and several other cancer types (Kaplan–Meier Plotter, http://kmplot.com/analysis). Taken together, these data suggest that genome alterations due to lowered or loss of *CHEK2* and *JAK2* expression may exacerbate cancer progression and predict poor patient survival. Collectively, our findings are consistent with a model in which JAK2 participates in the regulation of mitotic spindle assembly through the phosphorylation of CHK2 at Y156, together safeguarding the genomic stability to suppress neoplastic progression (Fig. 7G).

## Discussion

The spindle assembly checkpoint is crucial for the prevention of uneven chromosome segregation and aneuploidy, a predicament that often leads to neoplastic transformation. CHK2 plays a role in safeguarding the mitotic or spindle assembly checkpoint; however, the underlying mechanism of CHK2 regulation remains obscure. Our studies here have unveiled a signaling axis driven by JAK2, which links CHK2 Y156 phosphorylation to proper spindle assembly via the checkpoint kinase Mps1.

Our discovery that JAK2 plays a role in spindle assembly checkpoint is intriguing as the better-known activity of JAK2 appears to be cytokine/hematopoietic receptor-mediated signaling;[46] however, its association with mitotic apparatus has also been previously reported [33]. For example, JAK2 V617F, an activating mutation, causes polycythemia vera, the most common form of myeloproliferative neoplasm (MPN) [47]. Unexpectedly, the deletion of JAK2 can also be detected in some human cancers with a frequency comparable to that of its mutations (Fig. S10A). However, the implication of JAK2 deletion in human cancers has rarely been explored. JAK2 has been reported to localize to the centrosome where it regulates microtubule release but not microtubule nucleation, and its deletion results in chromosome misalignment and missegregation [33], a phenotype that closely resembles those observed in CHK2 KO and Y156F HeLa cells. Notably, in some MPN patients, JAK2 is expressed in fusion with a centrosomal protein PCM1 [48–50] and can reside at the centrosome with readily detectable kinase activity, which is likely to result from the dimerization of the centrosomal protein. Bochtler et al. [51]. explored this further by artificially tagging several tyrosine kinases involved in MPN with a centrosomal targeting domain and found that centrosomal targeting, albeit activated kinase signaling, failed to elicit oncogenicity. These results point to the possibility that besides ligand-receptor binding, centrosomal localization could be an alternative means for activating tyrosine kinases such as JAK2.

The mechanism of JAK2 regulation by spindle disruption remains an enigma. The centrosomal protein ninein negatively regulates JAK2 upon their interaction [33]. JAK2 binds and phosphorylates α- and β-tubulin; however, the functional significance is unclear [52]. Whether the interplay of these proteins could be altered by nocodazole, thereby enhancing the activity of JAK2 as observed in this study (Fig. 3A), remains to be determined.

Similar to JAK2, CHK2 is associated with mitotic structures. Takada et al. [53]. reported that Drosophila Chk2/Mnk localizes to centrosomes, interkinetochore/centromere region, midbody, and pseudocleavage furrows, in addition to the nucleus. Furthermore, CHK2 can be observed at kinetochores [24] and centrosomes [35] in mammalian cells during mitosis. Using confocal microscopy, we were able to demonstrate colocalization of JAK2 and Y156-phosphorylated CHK2 at the centrosome during mitosis (Fig. 4). It is possible that, upon spindle disruption, enhanced JAK2 kinase activity (Fig. 3A) would lead to increased CHK2 Y156 phosphorylation (Fig. 1D), perhaps at the centrosome or the microtubule organization center. Failure to do so, for example, in CHK2 KO, CHK2 Y156F, and JAK2-inhibited cells would result in impaired spindle assembly checkpoint and genome instability. The fact that these defects can be rescued by reexpression of WT and Y156E but not the Y156F mutant, further implicates the importance of CHK2 Y156 phosphorylation and the contribution of JAK2.

Unlike the unified view on the impact of CHK2 deficiency on genome stability, the perspectives on the effect on mitotic exit and its downstream effector have been varied, as demonstrated in different studies. We focused on CHK2 pY156-Mps1 signaling and the kinetochore localization of the latter. However, the involvement of BRCA1 [21, 54] and Aurora B [24] has also been proposed. In this study, we observed that CHK2 knockout leads to an accelerated mitotic exit, which can be corrected by WT CHK2. Although this is in line with the findings of Petsalaki and Zachos [24], it is in stark contrast to a previous report by Stolz et al. [21]. stating that CHK2 deficiency delayed mitotic exit. Further investigation is needed to resolve these differences.

Given the role of JAK2 in maintaining genome stability and its positive correlation with patient survival, caution is warranted when using JAK2 inhibitors for treating myeloproliferative disorders. Beyond its efficacy in killing cancer cells, perhaps as equally important, its impact on normal proliferating tissues should also be carefully measured.

## Materials and methods

### Cell culture, treatment, and transfection

HeLa and HEK293T cells were maintained in DMEM (Hyclone) medium supplemented with 10% fetal bovine serum (Hyclone) and 1% pen-strep (Gibco 15140-122). The CHK2 knockout (KO) HeLa cell line was generated as described previously [17]. The CHK2 KO cells were transfected with constructs expressing myc-CHK2 (WT or Y156F)-FLAG to establish G418-resistant stable cell lines. These cells were regularly maintained in a medium containing 400 μg/mL G418. The normal human fibroblasts MRC5 and WI38 were maintained in MEM (Gibco) supplemented with 10% fetal bovine serum and 1% pen-strep. All cell lines were from ATCC.

For ionizing radiation treatment, cells were exposed to 8 Gy X-ray using the Torrex 150D inspection system (EG&G) and then incubated for 2 h. Where indicated, cells were treated with CHK2 inhibitor II (C3742, Sigma Aldrich) and JAK2 inhibitor IV (#420139, Calbiochem) at concentrations of 5 and 2.5 μM, respectively. For nocodazole treatment of HeLa and HEK293T (M1404, Sigma Aldrich), either 50 ng/ml (for protein analysis) or 25 ng/ml (for confocal microscopy) was used. For the mitotic arrest of normal fibroblasts WI38 and MRC5, 600 ng/ml of nocodazole was used for protein analysis and 500 n/ml was used for confocal microscopy. Turbofect (Thermo Scientific) and calcium phosphate method were used for transfection of HeLa and HEK293T cells, respectively.

### Cell synchronization

Exponentially growing cells were treated with 50 ng/mL nocodazole overnight to arrest the cells in the mitotic phase. Double thymidine (Sigma, T1895) block was performed as previously described [21] to arrest cells in the G1/early S phase. For synchronization of normal fibroblasts, sub-confluent cells were incubated in a medium containing 0.2% fetal bovine serum for 3 days before replating in a complete medium. At 4.5 h after replating, cells were either collected (G0/G1), or maintained in a medium containing 4 μM aphidicolin overnight. Cells were then collected without release (G1/S) or released into a normal medium and collected 4 (S) or 8 (G2/M) hr after. The cell cycle was confirmed by flow cytometry using Attune N_X_T (Thermo Fisher).

### RNA interference

Sequences targeted by JAK2-1, JAK2-3, and CHK2 siRNA were 5′-CAAGATGTGAACTGTTTCT-3′, 5′-CGGATAGATCACATAAAAC-3′, and 5′-GTTGTTGGTAGTGGATCCA-3′, respectively. All siRNAs were synthesized by Sigma-Aldrich and transfected using Oligofectamine (Invitrogen) for 2 d before lysate collection.

### Plasmids

The construction of plasmids for mammalian and bacterial expression of CHK2 and Mps1 has been described previously [15, 16]. CHK2 Y156F was generated using site-directed mutagenesis coupled with PCR and confirmed by sequencing. The construction of mammalian expression plasmids for full-length and the C-terminal kinase domain of JAK2 has been described previously [55]. These plasmids were gifted by J. J. Yen (IBMS, Academia Sinica). For bacterial expression of His-JAK2-C (amino acids 843–1122), the corresponding cDNA was amplified from IMR90 cells, confirmed by sequencing, and cloned between the BamH I and EcoR I sites of pRSET A (Invitrogen).

### Purification of recombinant proteins

*E. coli* strain BL21 (DE3) Lys (Novagen) was transformed with plasmids and grown to OD_600_ 0.7– 0.9. The culture was then cooled and induced with IPTG (0.25–1 mM) for 2–3 h at 16–22 °C. After induction, the bacterial culture was collected, and the pellet was stored at −80 °C. Sonication buffer (50 mM NaH_2_PO_4_ pH 8, 0.3 M NaCl, 20% glycerol, 1 mM DTT, 10 mM β-mercaptoethanol, and 1 mM PMSF) was used to resuspend the pellets for sonication. Purification of His-tagged and GST-tagged recombinant proteins was performed as previously described [11] using either Ni-NTA Agarose (Qiagen) or Glutathione Sepharose 4B (GE Healthcare Biosciences).

### Cell lysis, immunoprecipitation, and western blot analysis

Whole-cell extracts were prepared by resuspending cells in TEGN buffer (10 mM Tris pH 7.5, 1 mM EDTA, 400 mM NaCl, 10% glycerol, and 0.5% NP-40) supplemented with a protease inhibitor cocktail (#04693132001, Roche Applied Science), 10 mM NaF, 10 mM β-glycerophosphate, 1 mM Na_3_VO_4_, and 1 mM DTT. For immunoprecipitation (IP), lysates were diluted with an equal volume of TEG buffer (10 mM Tris pH 7.5, 1 mM EDTA, and 20% glycerol) and rotated with the addition of control or specific antibodies at 4 °C for 1.5 h. Protein G (#53126, Thermo Scientific) or protein A beads (#20333, Thermo Scientific) were then added, and the mixtures were rotated for another 30 min. Beads were washed twice with IP buffer and once with PBS. Samples were boiled in protein sample buffer (166 mM Tris HCl pH 6.8, 4% SDS, 0.7 M 2-mercaptoethanol, 10% glycerol, and 1 mg/mL bromophenol blue) and subjected to gel electrophoresis. Proteins were transferred to nitrocellulose membranes, and immunoblot analysis was performed as described previously [17]. Chemiluminescence reagents (PerkinElmer) were used for the detection.

For co-immunoprecipitation of CHK2 and JAK2, cells were lysed in CSK buffer (10 mM PIPES pH 7.0, 100 mM NaCl, 3 mM MgCl_2_, 300 mM sucrose, and 0.1% Triton-X100) followed by brief sonication. After centrifugation, the supernatant was used directly for IP with either CHK2 or JAK2-specific antibodies.

In some western blots (Fig. 2A and Fig. S6D), lysates were prepared using RIPA buffer (50 mM HEPES, 1% NP-40, 0.5% sodium deoxycholate, 150 mM NaCl, 1 mM EDTA, and 0.1% SDS) instead.

### Antibodies

The antibodies used in this study are listed in Table S3. Rabbit polyclonal antibodies against phospho-Tyr156 were produced by LTK Biolaboratories (Touyuan, Taiwan) against the phosphopeptide H-CGPKNSpYIAYIED-OH synthesized by Kelowna International Scientific, Taiwan. The antibodies were affinity-purified by binding to a phosphopeptide column. The eluted antibodies were then passed through an unphosphorylated peptide column to remove antibodies that cross-reacted with unphosphorylated epitopes.

### In vitro kinase assays

For the SRC kinase assay, purified recombinant His-CHK2 WT or His-CHK2 Y156F proteins were incubated with recombinant SRC (#14-326, Millipore) in a kinase buffer containing 20 mM Tris pH 7.5, 10 mM MgCl_2_, 2 mM MnCl_2_, 1 mM DTT, 3 μM Na_3_VO_4_, and 0.25 μM ATP) for 30 min at 30 °C. The kinase reaction was stopped by boiling in the SDS loading dye. The reaction was analyzed by immunoblotting using phospho-Tyr and anti-CHK2 antibodies.

To compare the intrinsic kinase activity of CHK2 WT and Y156F, recombinant GST-cdc25A (amino acids 101–140) was used as the substrate, and the kinase reaction was performed in vitro as previously described [17].

For JAK2 kinase reaction, His-JAK2-C (bacterial expressed recombinant protein) or FLAG-JAK2-C that was immunoprecipitated from HEK-293T cells was incubated with His-CHK2 in a buffer containing 60 mM HEPES pH 7.5, 3 mM MgCl_2_, 3 mM MnCl_2_, 1.2 mM DTT, 3 μM Na_3_VO_4_, and 0.25 μM ATP for 20 min at 30 °C.

### Immunofluorescence microscopy

To analyze mitotic abnormalities, confocal microscopy was employed as previously described [33] with some modifications. Briefly, cells grown on coverslips were fixed with cold methanol for 10 min and then permeabilized with 0.5% Triton X-100 in PBS for 10 min at room temperature. Blocking was performed with 5% BSA in PBS, followed by overnight incubation with primary antibody. DAPI and the secondary antibody were used together at 4 °C for 1 h. Confocal images were captured with a confocal microscope (LSM700, Carl Zeiss). Abnormalities including misaligned metaphase chromosomes, lagging chromosomes, anaphase bridge formation, and micronuclei were examined in a range of 40–50 cells.

To assess Mps1 kinetochore localization, pre-extraction was performed with 0.05–0.1% Triton X-100 in PEM (100 mM PIPES, pH 6.8, 1 mM MgCl_2_, and 5 mM EGTA) for 1 min on ice before fixation with 4% paraformaldehyde in PBS for 10–15 min. Samples were then blocked with 3% BSA in PBS with 0.5% Triton X-100 for 30–35 min at room temperature and processed for antibody detection as described above.

Image analysis (co-localization or intensity) was performed using Zen Black 3.0 SR.

### Analysis of micronuclei

HeLa cells were seeded in 60 mm dishes and grown in a 5% CO_2_ incubator at 37 °C for 2 days before the addition of 50 ng/mL nocodazole. After overnight (~16 h) treatment, mitotic cells were collected via shake-off and washed twice with PBS. Cells were reseeded on coverslips in a complete medium and incubated for 2 days prior to fixation with 2% paraformaldehyde overnight. To detect micronuclei, samples were stained with DAPI, and images were captured using a Carl Zeiss LSM700 stage confocal microscope.

### Time-lapse live imaging

Mitotic cells were collected via shake-off after overnight nocodazole treatment. Cells were briefly incubated with NucBlue Live ReadyProbes reagent (Thermo Fisher R37605) for 20 min at 37 °C to stain the nucleus. After two washes with PBS, cells were reseeded in 96-well plates in media without phenol red and subjected to live imaging using Image Xpress^®^ Micro XL (Molecular Devices). Fluorescent images were captured every 10 min for 3–4 h. For comparison of the time between metaphase and cytokinesis, at least 20 cells were traced for each clone.

### Statistical analysis

All experiments were performed in triplicates. Data are expressed as mean ± SD. The student’s *t*-test was used to analyze the data unless specified otherwise. Differences were considered significant if *P* < 0.05.

## Supplementary information

supplemental information

Supplementary movie_parental

Supplementary movie_KO

Supplementary movie_WT

Supplementary movie_Y156F

uncropped images

reproducibility checklist

## Data Availability

The datasets are publicly available and were analyzed using the c-BioPortal for Cancer Genomics (https://www.cbioportal.org/) and the Kaplan–Meier Plotter (https://kmplot.com/analysis/) platforms.
