# Supplementary material for: JAK2-CHK2 signaling safeguards the integrity of the mitotic spindle assembly checkpoint and genome stability
Source: Cell Death Dis. 2022 Jul 18;13(7):619. doi: 10.1038/s41419-022-05077-0 (PMC9293949; doi:10.1038/s41419-022-05077-0)

Supplemental Information

JAK2-CHK2 signaling safeguards the integrity of the mitotic spindle assembly checkpoint

Chowdhury et al.

Supplemental Table S1-S3

Supplemental Figures and Figure Legends

Fig. S1 Related to Fig. 1

Fig. S2 Related to Fig. 1 and Fig. 3

Fig. S3 Related to Fig. 2

Fig. S4 Related to Fig. 3

Fig. S5 Related to Fig. 3

Fig. S6 Related to Fig. 4

Fig. S7 Related to Fig. 5

Fig. S8 Related to Fig. 5

Fig. S9 Related to Fig. 5

Fig. S10 Related to Fig. 7

Table 1. Microarray and RNA-seq data analyzed in this study			
Microarray data[a]		RNA-seq data[b]	
Cell line	Accession	CCLE[c] Name	DepMap ID
HCT116	GSE11618	Hela_CERVIX	ACH-001086
HEK293	GSE63862	HCT116_LARGE_INTESTINE	ACH-000971
Hela	GSE73055	KMRC1_KIDNEY	ACH-000684
		ACHN_KIDNEY	ACH-000046
		SIHA_CERVIX	ACH-000556

[a] Array platform: Affymetrix Human Genome U133 Plus 2.0 Array

[b] Public release version: CCLE_depMap_18Q4_TPM_v2

[c] Cancer Cell Line Encyclopedia

Table 2. Tyrosine kinases co-expressed with CHK2 with Spearman's correlation coefficient > 0.7 and p-value < 0.05 in RNA-seq and microarray datasets

Kinase	correlation	p-value	Subcellular location
RNA-seq			
WEE1	0.939	0.018	Nucleus
EPHA1	0.924	0.025	Plasma membrane
JAK2	0.921	0.026	Nucleus, other locations
FES	0.894	0.041	Plasma membrane, cytosol, golgi apparatus, other locations
RET	0.888	0.044	Plasma membrane, endosome
Microarray			
EPHA5	0.978	2.76E-05	Plasma membrane, other locations
ALK	0.977	2.96E-05	Plasma membrane
IGF1R	0.974	4.44E-05	Plasma membrane
CSK	0.970	6.76E-05	Plasma membrane, other locations
FAK	0.962	1.35E-04	Plasma membrane, cytoskeleton, nucleus, other locations
WEE1	0.959	1.63E-04	Nucleus
ZAP70	0.929	8.55E-04	Plasma membrane, other locations
NTRK3	0.926	9.77E-04	Membrane
EPHA6	0.922	1.12E-03	Membrane
PDGFRB	0.906	1.94E-03	Plasma membrane, lysosome, other locations
PDGFRA	0.905	2.02E-03	Golgi apparatus, nucleus, plasma Membrane, other locations
EPHA3	0.905	2.02E-03	Plasma membrane, extracellular region or secreted
FYN	0.893	2.80E-03	Nucleus, plasma membrane, other locations
NTRK2	0.863	5.78E-03	Plasma membrane, endosome, other locations
EPHA10	0.845	8.19E-03	Plasma membrane, extracellular region or secreted
EGFR	0.815	0.014	Plasma membrane, nucleus, endosome, golgi apparatus, ER
LYN	0.801	0.017	Plasma membrane, nucleus, golgi apparatus, other locations
SRC	0.759	0.029	Plasma membrane, nucleus, mitochondrion, cytoskeleton, other locations
INSR	0.721	0.044	Plasma membrane, endosome, lysosome

Table S3. List of antibodies

Antibodies	Source	Identifier	Dilution	Application
JAK2	Cell Signalling	3230	1:1000<br>4-500 ng<br>1:100	WB<br>IP<br>IF
	Everest Biotech	EB05075	1:100	IF
Phospho-JAK2 (Tyr1007/1008)	Cell Signalling	3771	1:500	WB
CHK2	MBL	K0087-3	1:1000	WB
	Santa Cruz	sc-8813	1:1000<br>500 ng<br>1:100	WB<br>IP<br>IF
	Bethyl	A300-619A	500 ng	IP
	Cell Signalling	3440	1:500	WB
Phospho-CHK2 (Thr68)	Cell Signalling	2197	1:1000	WB
Myc tag	Santa Cruz	sc-40	1:500	WB
	Bethyl	A190-104A	4-500ng	IP
His tag	LTK BioLaboratories		1:1000	WB
HA tag	LTK BioLaboratories		1:1000	WB
FLAG tag	LTK BioLaboratories		1:1000	WB
GST	Santa Cruz	sc-138	1:1000	WB
Phospho-Tyrosine	Invitrogen	61-5800	1:1000	WB
	Cell Signalling	9411	1:500	WB
Cdc25A	Abcam	ab 63391	1:500	WB
Phospho-Cdc25A (Ser124)	Abcam	ab63391	1:500	WB
Histone H3	Millipore	07-690	1:10000	WB
Phospho-Histone H3 (Ser10)	Millipore	06-570	1:500	WB
	GeneTex	GTX61067	1:500	WB
Cyclin A	BD Bioscience Pharmingen	611268	1:500	WB
Cyclin B1	Santa Cruz	sc-245	1:500	WB
TTK/hMps1	Upstate	05-683	1:500<br>1:50	WB<br>IF
	Bethyl	A300-296A	500 ng	IP
β-Actin	GeneTex	GTX109639	1:1000	WB

α-Tubulin	Sigma	T6074	1:100	IF
γ-Tubulin	Sigma	T6557	1:200	IF
Phospho-CHK2 (Ser516)	Cell Signalling	2669	1:500	WB
CENP-B	Santa Cruz	sc-22788	1:100	IF
Akt	BD	610860	1:500	WB
Phospho-ERK1&2 (pTpY185/187)	Invitrogen	44680G	1:500	WB
Phospho-Akt (S473)	Cell Signalling	4060S	1:500	WB
HEC1	GeneTex	GTX70268	1:500	WB
HRP-anti-rabbit IgG	Bethyl	A120-201P	1:10000	WB
HRP-anti-mouse IgG	Bethyl	A90-516P	1:10000	WB
HRP-anti-goat IgG	Bethyl	A50-201P	1:10000	WB
HRP-anti-rabbit IgG light chain	Jackson ImmunoResearch	211-032-171	1:5000	WB
FITC-anti-mouse IgG	Jackson ImmunoResearch	115-095-146	1:1-300	IF
TRITC-anti-mouse IgG	Jackson ImmunoResearch	115-025-146	1:1-300	IF
TRITC-anti-rabbit IgG	Jackson ImmunoResearch	111-026-003	1:100-300	IF
Cy5-anti-goat IgG	Jackson ImmunoResearch	705-175-147	1:100-300	IF
CHK2 pY156	House-generated		1:100<br>1:500	IF<br>WB

Figure S1. Identification of mitosis-related CHK2 phosphorylation sites by mass spectrometry. (A) Summary of identified phosphorylation sites. Ectopically expressed myc-CHK2 was immunoprecipitated from transfected HEK293T cells with or without overnight nocodazole treatment and then analyzed by mass spectrometry. Those marked in red (Y156, S194, T441, and T477) are sites only found in treated cells while those marked in green (S187 and S500) are only observed in untreated cells. (B) MS spectrum of Y156-phosphorylated peptide. (C) Cross-species alignment showing conservation of Y156 and surrounding amino acids.

Figure S2. JAK2 phosphorylates CHK2 on Y156. (A) Dot blots showing that the CHK2 pY156-specific antibody reacted with Y156-phosphorylated peptide but not with the corresponding unphosphorylated peptide. (B) Nocodazole induced CHK2 Y156 but not T68 phosphorylation in HeLa cells. Whole cell lysates were analyzed by western blotting using the purified CHK2 pY156-specific antibody. CHK2 KO cells were used as negative control. (C) CHK2 Y156 phosphorylation was dampened in JAK2 knockdown cells. HeLa cells were transfected with JAK2 siRNA JAK2-1 or JAK2-3 for 2 d then treated with nocodazole overnight. “*” indicates non-specific band. (D, E) JAK2-C specifically phosphorylated CHK2 at Y156. *In vitro* kinase assays were performed by incubating FLAG-JAK2-C immunoprecipitated from transfected HEK293T cells with either recombinant GST-CHK2-FHA domain (D) or recombinant full-length (FL) His-CHK2 (E) as substrate. Phosphorylation was detected in WT but not Y156F CHK2, which can be inhibited by JAK2 inhibitor IV. (F) JAK2 inhibition does not hamper JAK2-CHK2 interaction. Endogenous JAK2-CHK2 co-immunoprecipitation was performed using HeLa WT CHK2 clone #103 that has been treated with nocodazole overnight. Cells were treated with JAK2 inhibitor for 2 h prior to collection.

Figure S3. Assessment of chromosome alignment by confocal microscopy. (A) Mitotic progression following release from nocodazole. Chromosomes were stained with DAPI, and mitotic spindles were stained with anti-α-tubulin. Images from parental HeLa cells are shown. Scale bar, 10 μM. (B) Representative images of proper and improper nocodazole-arrested cells. (C) Representative images of normal and abnormal metaphase chromosomes.

Figure S4. **Bioinformatics approach for identifying potential CHK2-targeting tyrosine kinases.** (A) Flow chart for the bioinformatics analysis performed to identify tyrosine kinases that target CHK2. (B) Pairwise correlation plot of co-expression between CHK2 and 41 tyrosine kinases derived from RNA-seq data in hierarchical clustering order. Positive and negative correlations are shown in blue and red, respectively. Circle size is scaled to the adjusted *P*-value, the larger the size the lower *P*-value. *P*-values higher than 0.5 are left blank. Matrix cells with *P*-value < 0.05 for kinases co-expressed with CHK2 are boxed in green.

Figure S5. CHK2 is not a substrate of WEE1, SRC, or EGFR. (A) WEE1 may not be a CHK2 tyrosine kinase. HEK293T cells were co-transfected with HA-tagged WEE1 (HA-WEE1) and myc-CHK2, and tyrosine phosphorylation of CHK2 was assayed by western blot following immunoprecipitation of myc-CHK2. Cells were treated with 1 mM pervanadate for 10 min before collection. (B) SRC did not efficiently phosphorylate CHK2 under *in vitro* conditions. Kinase reaction was performed with recombinant SRC and purified His-CHK2 FL in the presence or absence of ATP, and phosphorylation was assessed with western blot performed using the anti-pTyr antibody. (C) EGF did not induce CHK2 tyrosine phosphorylation.

HeLa cells were serum-starved overnight and then treated with 10 ng/mL EGF for 5 min. Lysates were collected for immunoblot analysis performed using the indicated antibodies. The results indicated that EGFR may not be a CHK2 kinase. AKT phosphorylation was used as a positive control for EGF treatment.

Figure S6. CHK2 pY156 and JAK2 colocalize at centrosomes in mitotic normal fibroblasts. MRC5 cells were transfected with control (sc) or CHK2 siRNA for 2 d and then treated with 500 ng/ml nocodazole overnight (prometaphase, PM) followed by release into normal medium for 1 h (metaphase, M). Cells were processed for confocal microscopy using the indicated antibodies (A). Images and colocalization at centrosomes were analyzed and is shown in (B). Western blots show effective knockdown of CHK2 (C). Scale bar, 5 μM.

Figure S7. The intrinsic kinase activity of CHK2 Y156F was intact. (A) His-CHK2 WT and Y156F phosphorylated GST-CDC25A (amino acids 101-140) equally under *in vitro* conditions. Phosphorylation was detected with western blot performed using the anti-CDC25A pS124-specific antibody. (B, C) His-CHK2 WT and Y156F could autophosphorylate S516 (B) and T68 (C) under *in vitro* conditions.

Figure S8. Mps1 knockdown control for Mps1 and CENP-B colocalization staining. (A) Mps1 siRNA efficiently downregulated Mps1 expression in HeLa WT and Y156F CHK2 stable clones. (B) Mps1 kinetochore localization was not detected in Mps1 knockdown cells; however, it was detected in control cells (Fig. 5C). The results indicated the specificity of the Mps1 staining. Scale bar, 10 μM.

Figure S9. CHK2 T68 phosphorylation is not involved in the spindle assembly checkpoint. (A) CHK2 in nocodazole-treated HeLa cells did not harbor T68 phosphorylation. Cells irradiated with X-ray and collected 1 h after served as the positive control. (B) Phosphorylation at T68 and Y156 are independent of each other. The CHK2 T68A mutant can still be phosphorylated by JAK2-C at Y156 in vitro. (C, D) CHK2 T68A can interact with (C) and phosphorylate Mps1 at T288 (D). Immunoprecipitation was performed using HEK293T lysates coexpressing HA-Mps1 and myc-CHK2. (E) CHK2 interaction with HEC1 is not affected by the T68A mutation. Co-immunoprecipitation was performed using transfect HEK293T cells treated with nocodazole overnight. (F, G) The stability of Mps1 is similar when coexpressed with WT or T68A CHK2. Transfected HEK293T cells were treated with 50 μg/ml cycloheximide, collected at the indicated time points and analyzed by western blotting (F). Results from 4 independent experiments were quantified and are shown as mean ± SD in (G).

Figure S10. Pan-cancer analysis of the alteration and expression of *CHEK2* and *JAK2*. (A) Alteration of *JAK2* in metastatic cancers. *JAK2* alterations were analyzed using the cBioportal for Cancer Genomic (https://www.cbioportal.org) platform with published datasets (39). n = 10945. (B-E) Lower expression of *CHEK2* and *JAK2* is associated with poorer overall survival in patients with rectal adenocarcinoma (B, C) and ovarian cancer (D, E). Overall survival among patients was analyzed using Kaplan-Meier Plotter (http://kmplot.com/analysis/) on pan-cancer RNA-seq datasets. Shown are results obtained from the analysis of datasets derived from patients with rectal adenocarcinoma (n = 165) and ovarian cancer (n = 374) for the expression of *CHEK2* (B, D) and *JAK2* (C, E).

Figure S1

A

S120, S140, S155, Y156, Y159, S164, T168, S187, S192, S194, S223, T225, S228, S260, S356, S357, S372, T378, S379, T383, T387, T389, Y390, T401, T432, S435, T441, S456, T476, T477, S500, T506, S516, S518, T517

1 MSRESDVEAQ QSHGSSACSQ PHGSVTQSQG SSSQSQGISS SSTSTMPNSS QSSHSSSGTL

61 SSLETVSTQE LYSIPEDQEP EDQEPEEPTP APWARLWALQ DGFANLECVN DNYWFGRDKS

121 CEYCFDEPLL KRTDKYRTYS KKHFRIFREV GPKNSYIAYI EDHSGNGTFV NTELVGKGKR

181 RPLNNNSEIA LSLSRNKVFV FFDLTVDDQS VYPKALRDEY IMSKTLGSGA CGEVKLAFER

241 KTCKKVAIKI ISKRKFAIGS AREADPALNV ETEIEILKKL NHPCIIKIKN FFDAEDYYIV

301 LELMEGGELF DKVVGNKRLK EATCKLYFYQ MLLAVQYLHE NGIIHRDLKP ENVLLSSQEE

361 DCLIKITDFG HSKILGETSL MRTLCGTPTY LAPEVLVSVG TAGYNRAVDC WSLGVILFIC

421 LSGYPPFSEH RTQVSLKDQI TSGKYNFIPE VWAEVSEKAL DLVKKLLVVD PKARFTTEEA

481 LRHPWLQDED MKRKFQDLLS EENESTALPQ VLAQPSTSRK RPREGEAEGA ETTKRPAVCA

541 AVL

B

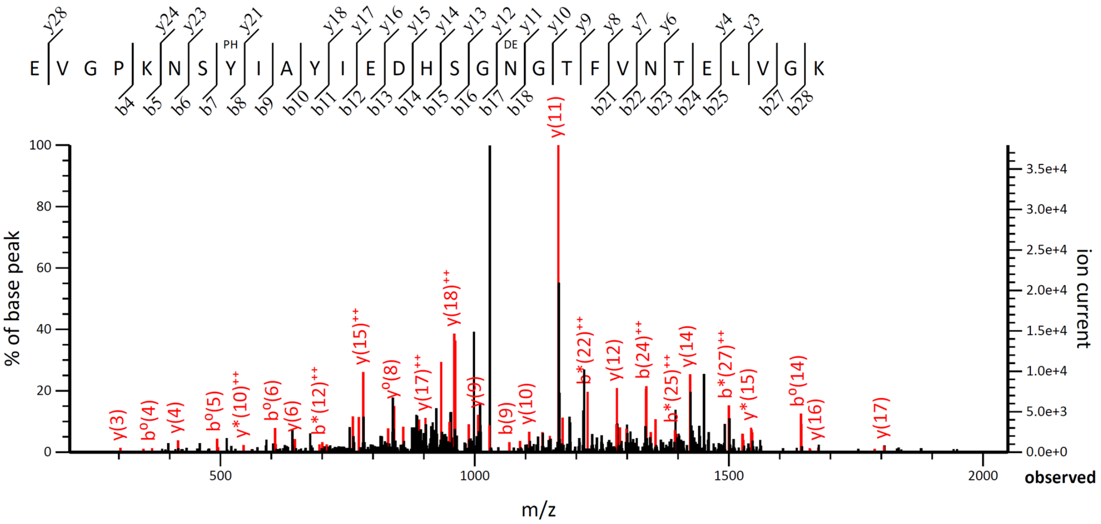

C

Species	Sequence	NCBI Reference Sequence
Mouse (Mus musculus)	GPKNCYIVYIE	NP_057890.1
Rat (Rattus norvegicus)	GPKNCYIVYIE	NP_446129.1
Dog (Canis lupus familiaris)	GPKNCYIAYIE	XP_005636447.1
Cattle (Bos Taurus)	GPKNSYIAYIE	NP_001029703.1
Chimpanzee (Pan troglodytes)	GPKNSYIAYIE	XP_003317205.1
Rhesus macaque (Macaca mulatta)	GPKNSYIAYIE	XP001101658.1
Human (Homo sapiens)	GPKNSYIAYIE	AAD48504.1
Red junglefowl (Gallus gallus)	GPKNSYIAYIE	NP_001073576.1

Y156

Figure S2

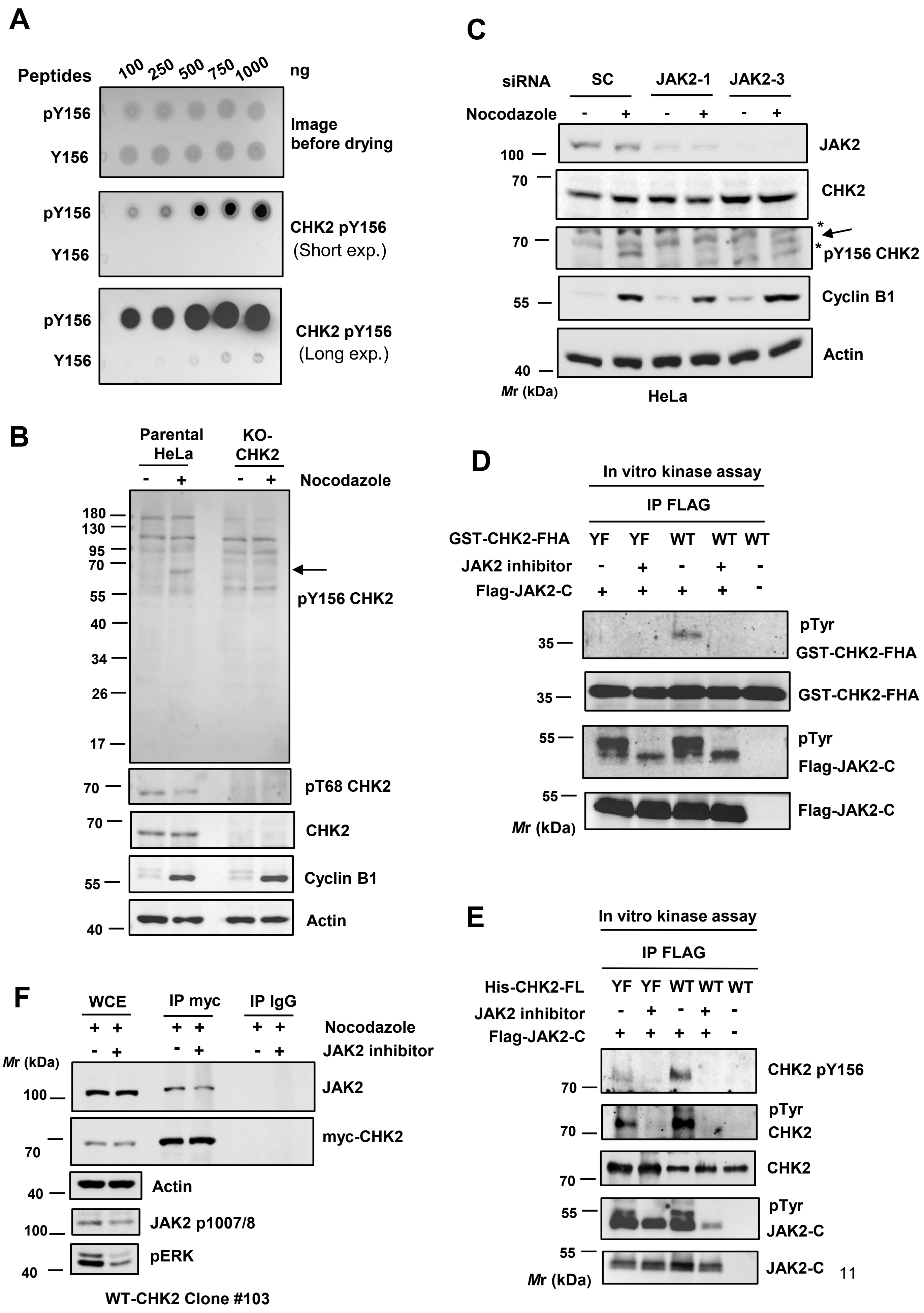
A
Peptides
100 250 500 750 1000 ng
pY156
Y156
Image before drying
CHK2 pY156
(Short exp.)
CHK2 pY156
(Long exp.)
B
Parental HeLa
KO-CHK2
- + - + Nocodazole
180
130
95
70
55
40
34
26
17
pY156 CHK2
pT68 CHK2
CHK2
Cyclin B1
Actin
C
siRNA SC JAK2-1 JAK2-3
Nocodazole - + - + - +
100
70
55
40
JAK2
CHK2
pY156 CHK2
Cyclin B1
Actin
Mr (kDa)
HeLa
D
In vitro kinase assay
IP FLAG
GST-CHK2-FHA YF YF WT WT WT
JAK2 inhibitor - + - + -
Flag-JAK2-C + + + + -
35
55
pTyr
GST-CHK2-FHA
GST-CHK2-FHA
pTyr
Flag-JAK2-C
Flag-JAK2-C
Mr (kDa)
E
In vitro kinase assay
IP FLAG
His-CHK2-FL YF YF WT WT WT
JAK2 inhibitor - + - + -
Flag-JAK2-C + + + + -
CHK2 pY156
pTyr
CHK2
CHK2
pTyr
JAK2-C
JAK2-C
70
55
Mr (kDa)
F
WCE IP myc IP IgG
+ + + + + + Nocodazole
- + - + - + JAK2 inhibitor
Mr (kDa)
100
70
40
JAK2
myc-CHK2
Actin
JAK2 p1007/8
pERK
WT-CHK2 Clone #103

Figure S3

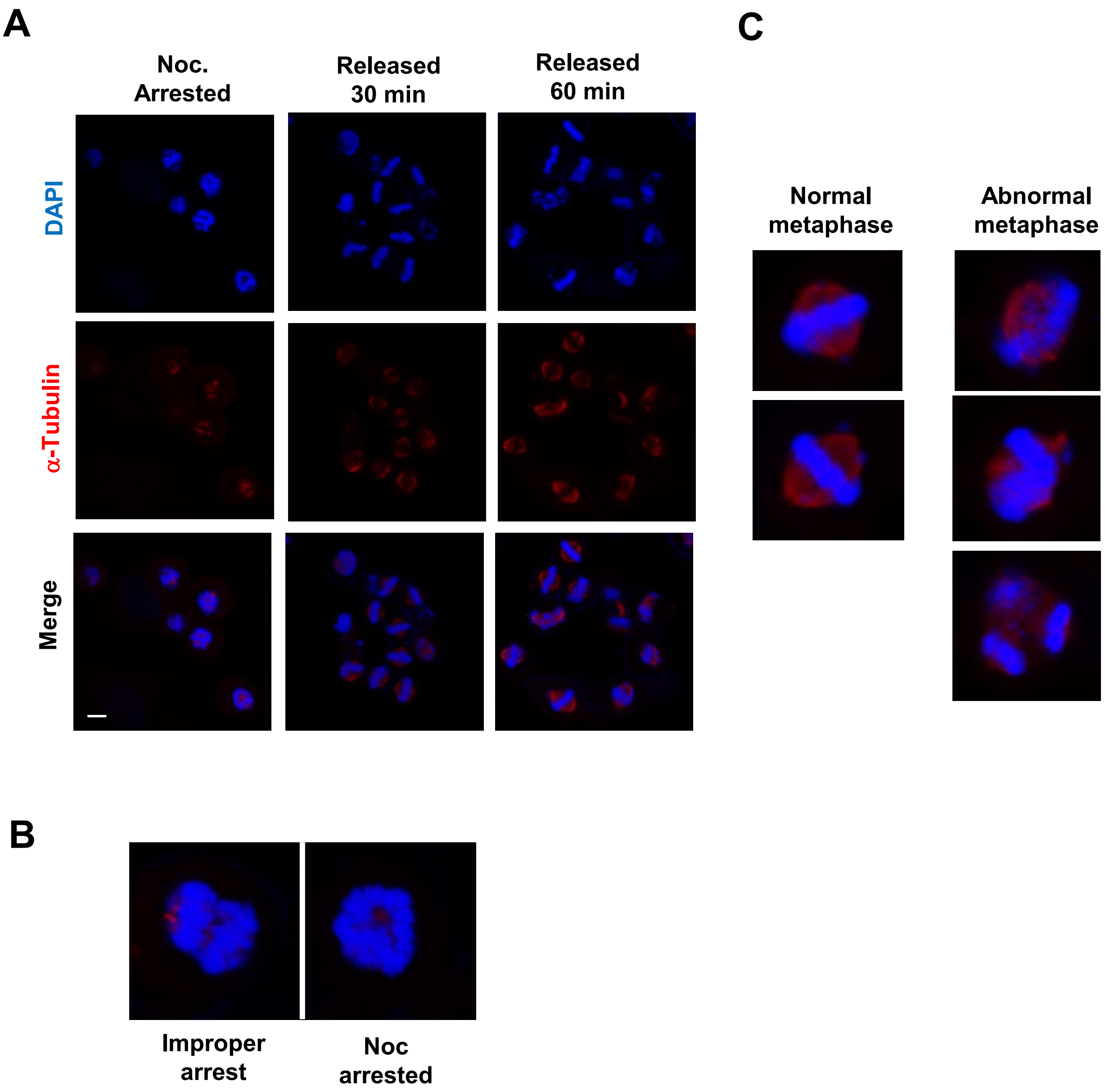
A
Noc.
Arrested
Released
30 min
Released
60 min
DAPI
α-Tubulin
Merge
B
Improper
arrest
Noc
arrested
C
Normal
metaphase
Abnormal
metaphase

Figure S4

A

41 coding genes for 26 Tyr kinases from KinasePhos2.0

Extract RNA-seq expression data from DepMap portal (5 cell lines)

Extract microarray expression data from NCBI GEO database (3 cell lines)

Pairwise Spearman’s rank correlation analysis for coexpression of CHK2 and Tyr kinase genes using Hmisc/R packages

Visualize correlation matrix with p-value < 0.5 using corrplot/R packages

B

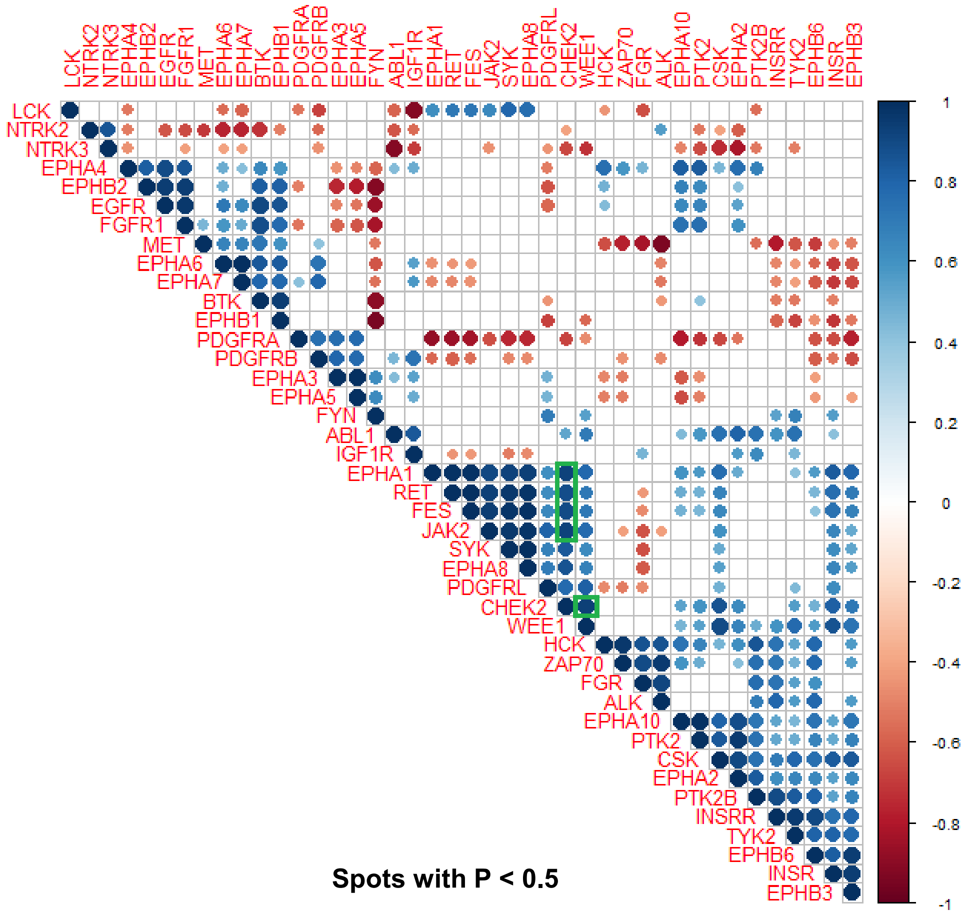

Spots with P < 0.5

Figure S5

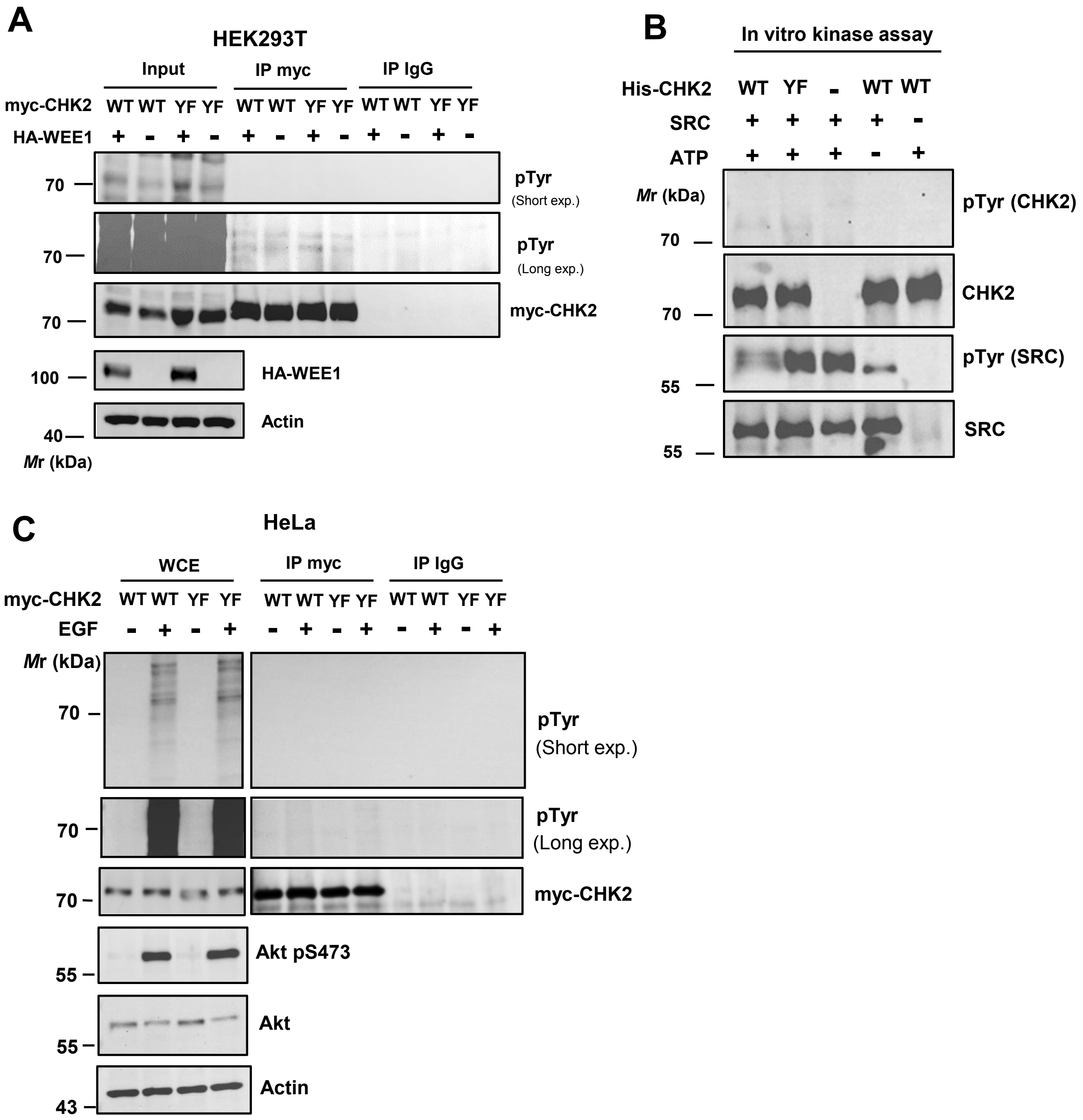

A
HEK293T
Input
IP myc
IP IgG
myc-CHK2 WT WT YF YF WT WT YF YF WT WT YF YF
HA-WEE1 + - + - + - + - + - + -
70
pTyr
(Short exp.)
70
pTyr
(Long exp.)
70
myc-CHK2
100
HA-WEE1
Actin
40
Mr (kDa)
B
In vitro kinase assay
His-CHK2 WT YF - WT WT
SRC + + + + -
ATP + + + - +
Mr (kDa)
pTyr (CHK2)
70
CHK2
70
pTyr (SRC)
55
SRC
55
C
HeLa
WCE
IP myc
IP IgG
myc-CHK2 WT WT YF YF WT WT YF YF WT WT YF YF
EGF - + - + - + - + - + - +
Mr (kDa)
70
pTyr
(Short exp.)
70
pTyr
(Long exp.)
70
myc-CHK2
Akt pS473
55
Akt
55
Actin
43

Figure S6

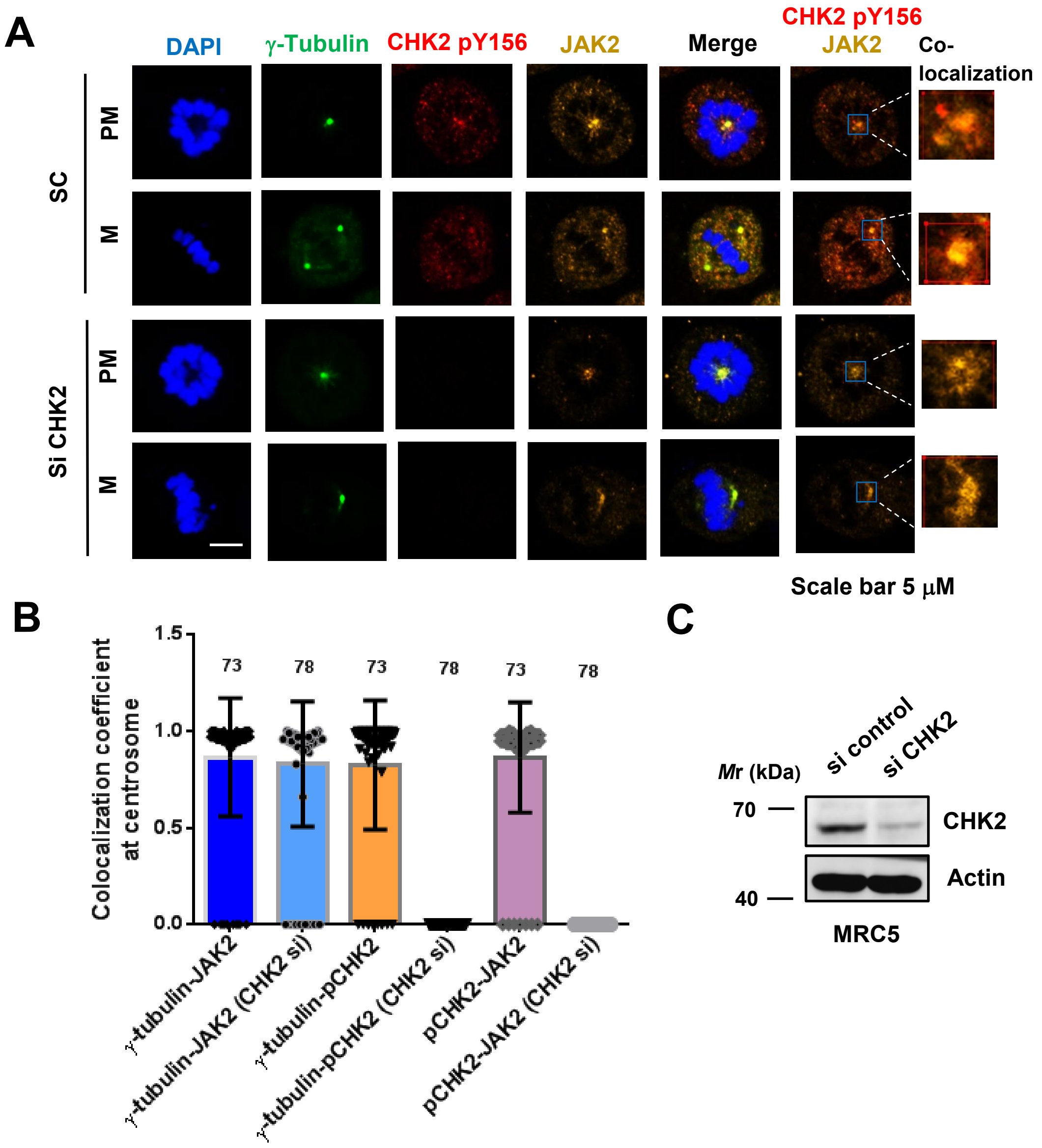
A
DAPI
γ-Tubulin
CHK2 pY156
JAK2
Merge
CHK2 pY156
JAK2
Co-localization
SC
PM
M
Si CHK2
PM
M
Scale bar 5 μM
B
Colocalization coefficient at centrosome
1.5
1.0
0.5
0.0
73
78
73
78
73
78
γ-tubulin-JAK2
γ-tubulin-JAK2 (CHK2 si)
γ-tubulin-pCHK2
γ-tubulin-pCHK2 (CHK2 si)
pCHK2-JAK2
pCHK2-JAK2 (CHK2 si)
C
Mr (kDa)
si control
si CHK2
70
40
CHK2
Actin
MRC5

Figure S7

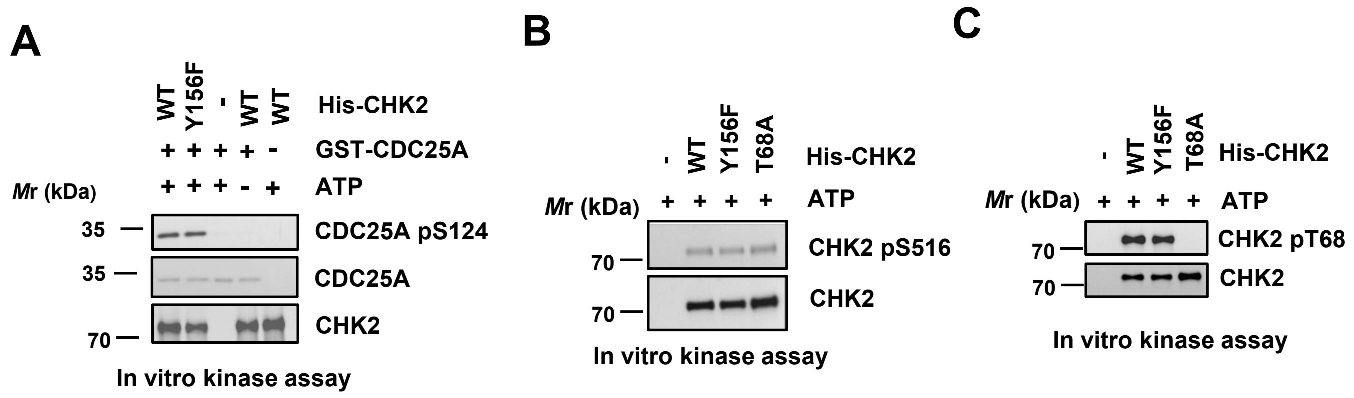
A
WT
Y156F
-
WT
WT
His-CHK2
+ + + + -
GST-CDC25A
Mr (kDa)
+ + + - +
ATP
35
CDC25A pS124
35
CDC25A
70
CHK2
In vitro kinase assay
B
-
WT
Y156F
T68A
His-CHK2
Mr (kDa)
+ + + +
ATP
70
CHK2 pS516
70
CHK2
In vitro kinase assay
C
-
WT
Y156F
T68A
His-CHK2
Mr (kDa)
+ + + +
ATP
70
CHK2 pT68
70
CHK2
In vitro kinase assay

Figure S8

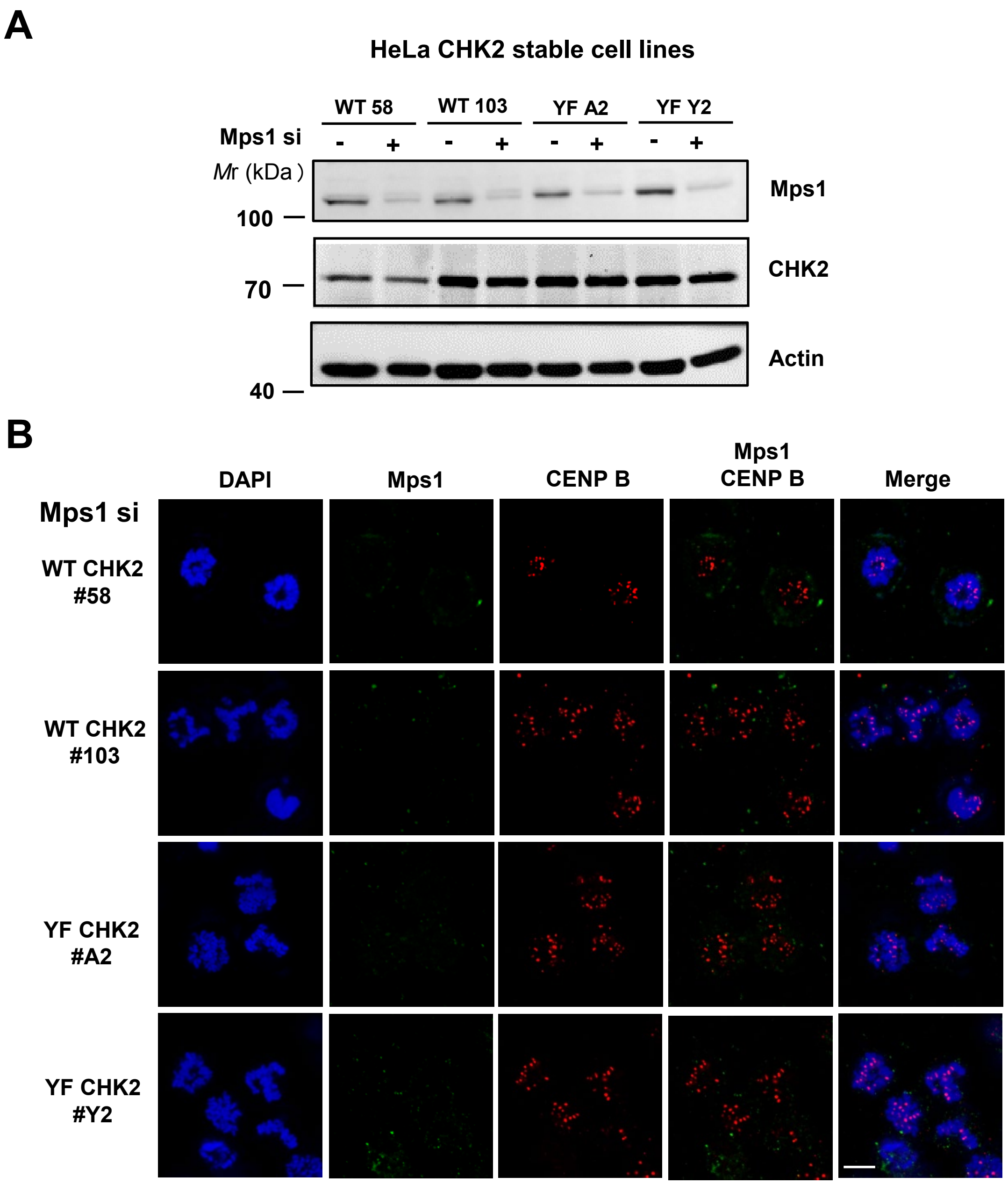
A
HeLa CHK2 stable cell lines
WT 58
WT 103
YF A2
YF Y2
Mps1 si
- + - + - + - +
Mr (kDa)
100
70
40
Mps1
CHK2
Actin
B
DAPI
Mps1
CENP B
Mps1
CENP B
Merge
Mps1 si
WT CHK2
#58
WT CHK2
#103
YF CHK2
#A2
YF CHK2
#Y2

Figure S9

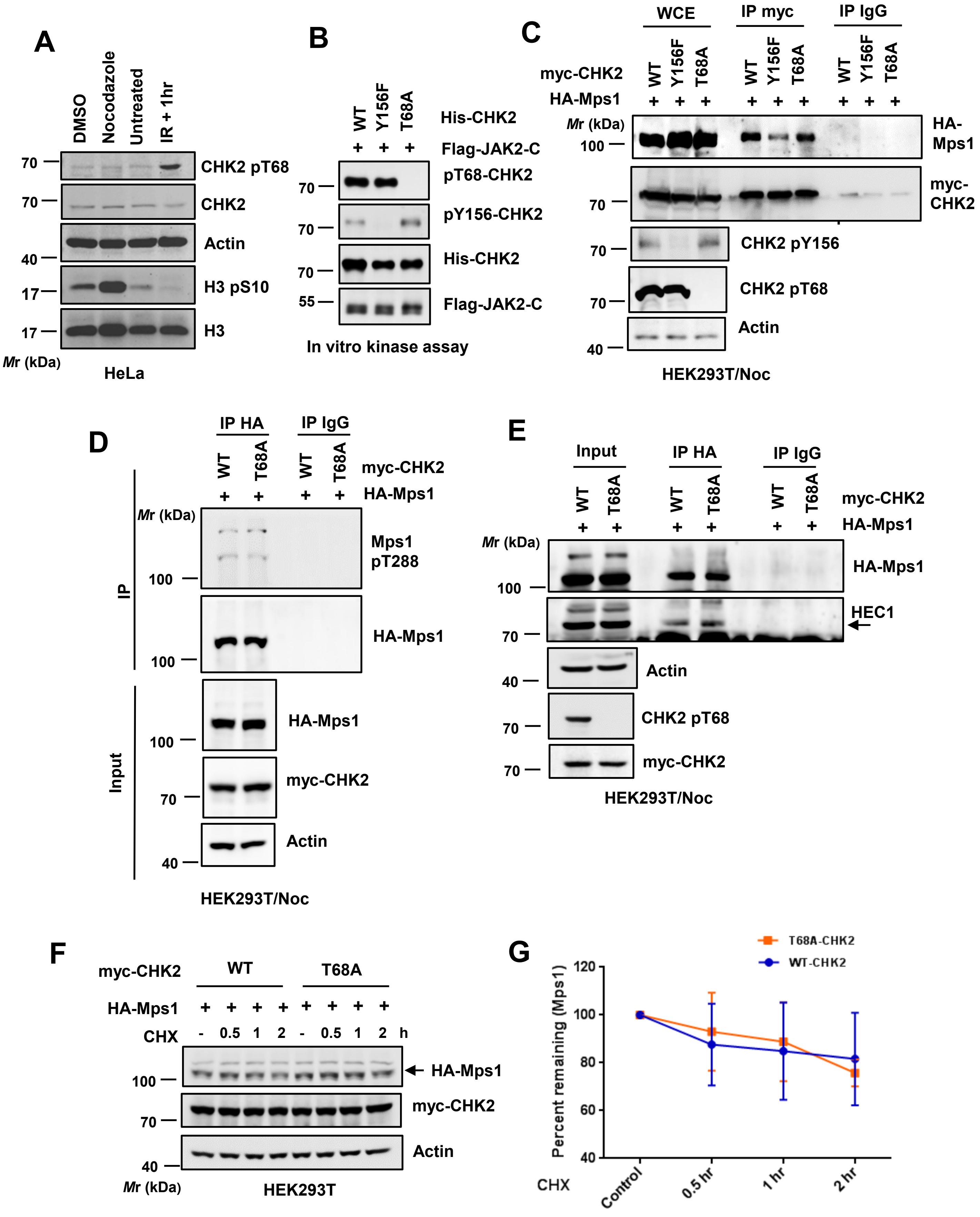
A
DMSO
Nocodazole
Untreated
IR + 1hr
CHK2 pT68
CHK2
Actin
H3 pS10
H3
Mr (kDa)
HeLa
B
WT
Y156F
T68A
His-CHK2
Flag-JAK2-C
pT68-CHK2
pY156-CHK2
His-CHK2
Flag-JAK2-C
In vitro kinase assay
C
WCE
IP myc
IP IgG
myc-CHK2
HA-Mps1
HA-Mps1
myc-CHK2
CHK2 pY156
CHK2 pT68
Actin
HEK293T/Noc
D
IP HA
IP IgG
Mps1 pT288
HA-Mps1
IP
Input
myc-CHK2
Actin
HEK293T/Noc
E
Input
IP HA
IP IgG
HA-Mps1
HEC1
Actin
CHK2 pT68
myc-CHK2
HEK293T/Noc
F
WT
T68A
CHX
HA-Mps1
myc-CHK2
Actin
HEK293T
G
T68A-CHK2
WT-CHK2
Percent remaining (Mps1)
CHX
Control
0.5 hr
1 hr
2 hr

Figure S10

A

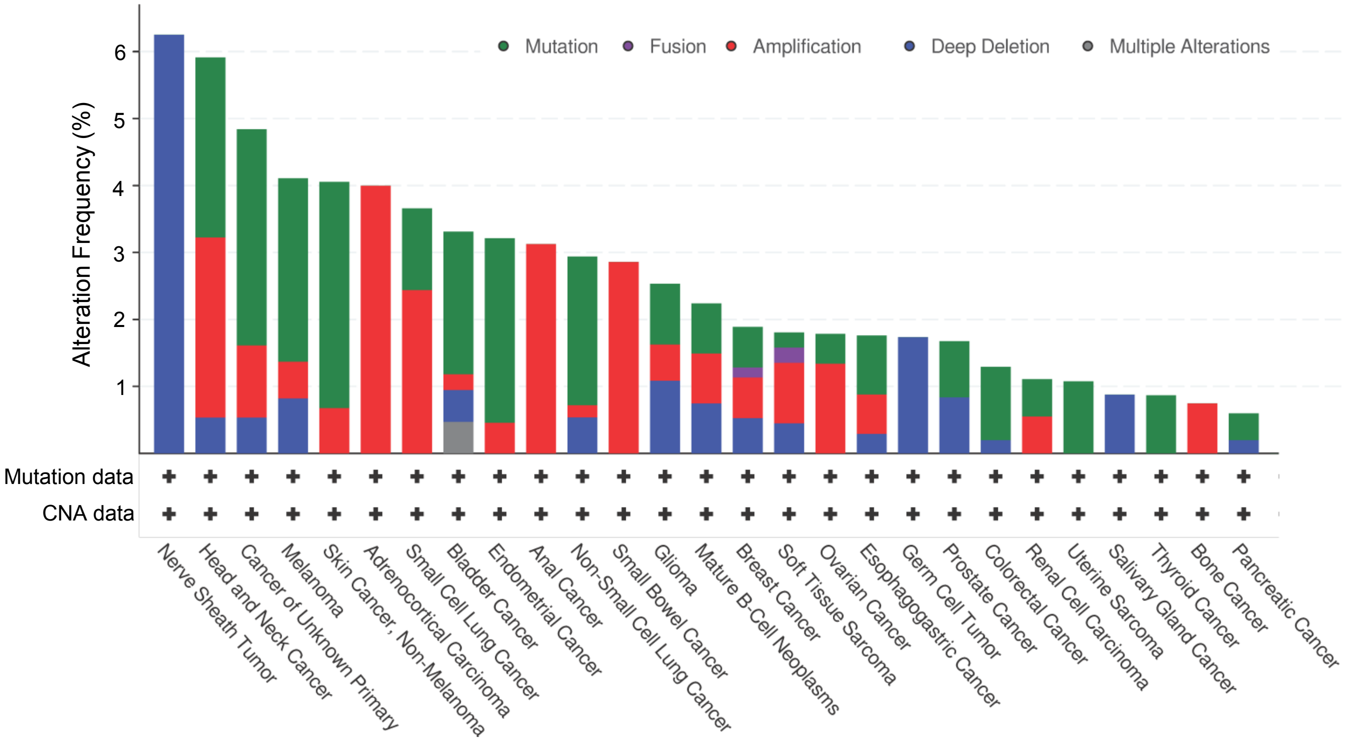

B

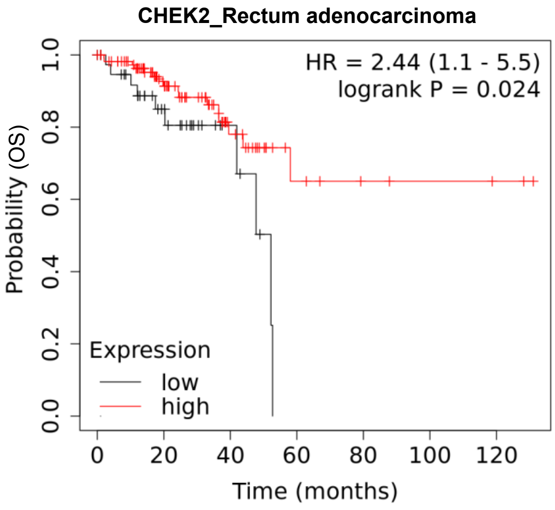

C

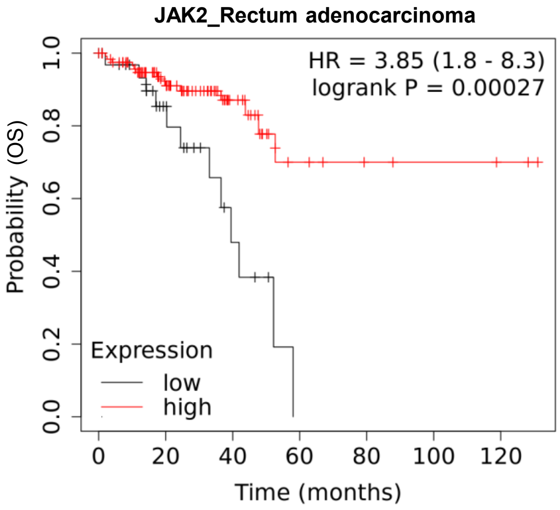

D

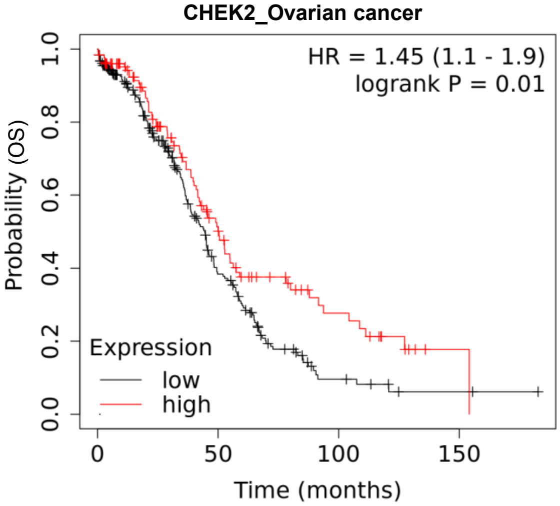

E

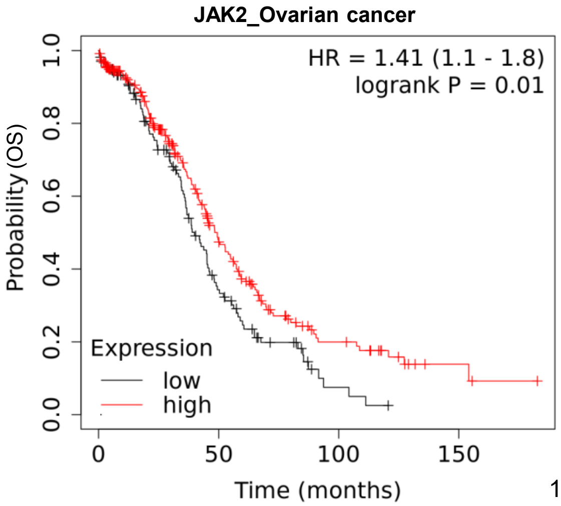

Supplement: Supplementary file 1 — supplemental information [file 41419_2022_5077_MOESM1_ESM.pdf]
