# Supplementary material for: JAK2-CHK2 signaling safeguards the integrity of the mitotic spindle assembly checkpoint and genome stability
Source: Cell Death Dis. 2022 Jul 18;13(7):619. doi: 10.1038/s41419-022-05077-0 (PMC9293949; doi:10.1038/s41419-022-05077-0)

Figure 1 A

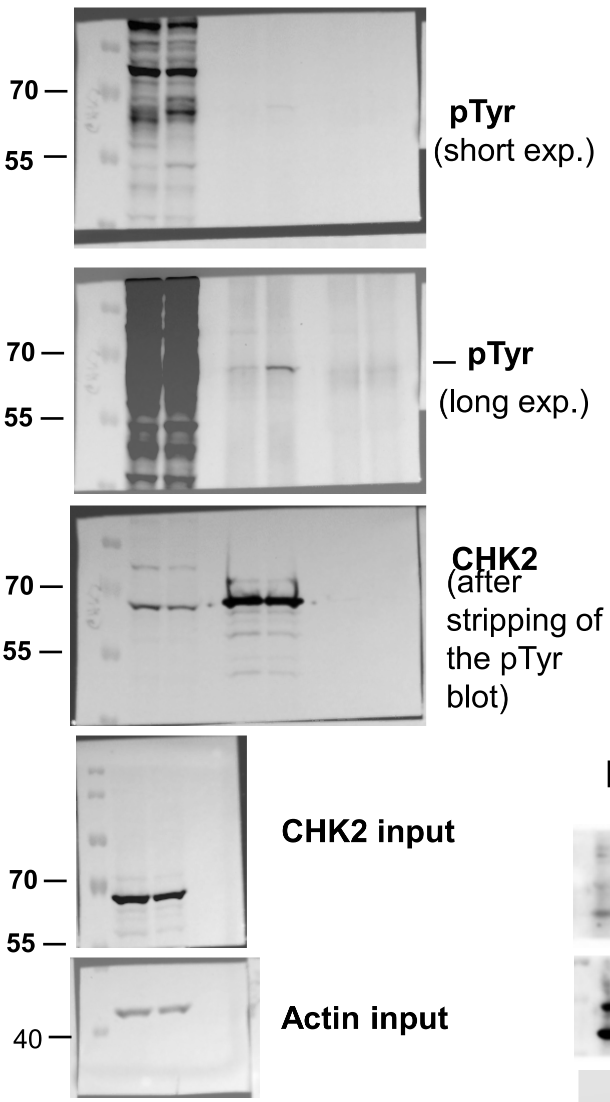

Figure 1 B

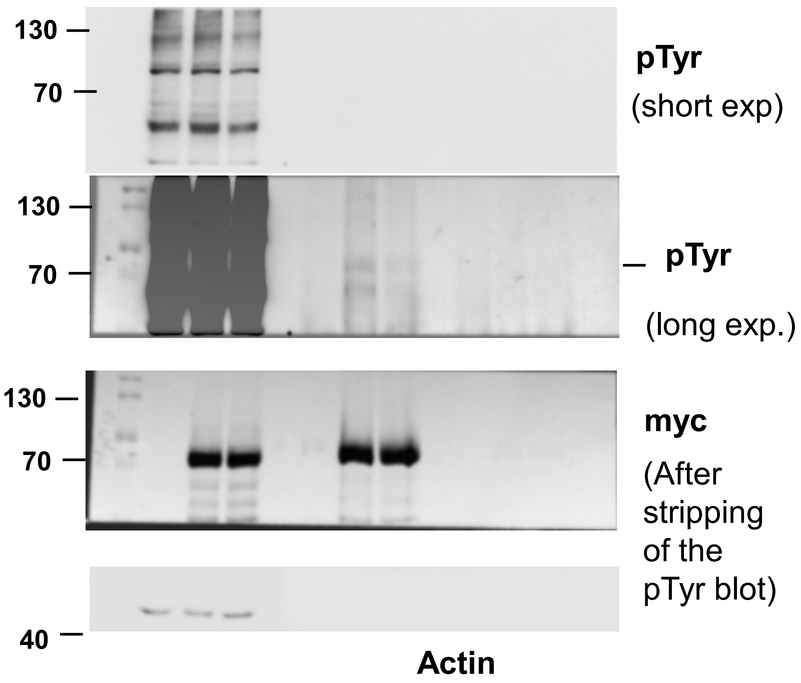

Figure 1 E

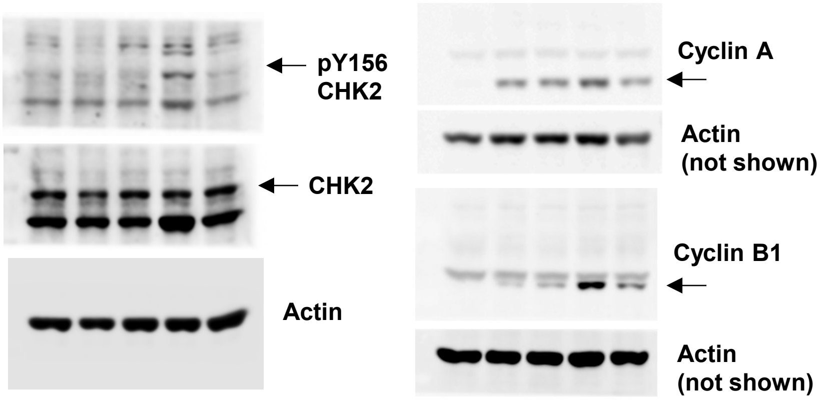

Figure 1 F

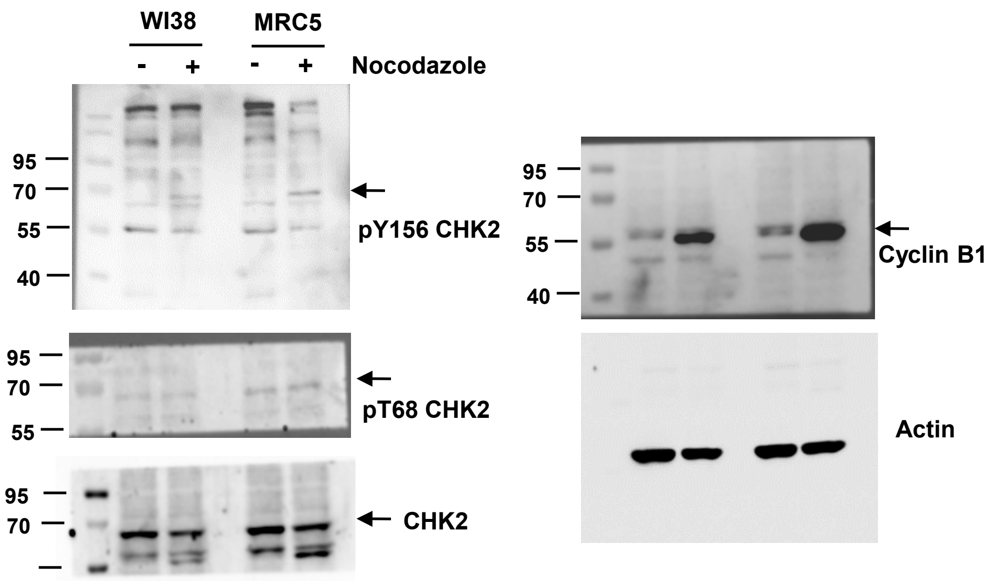

Figure 1 C

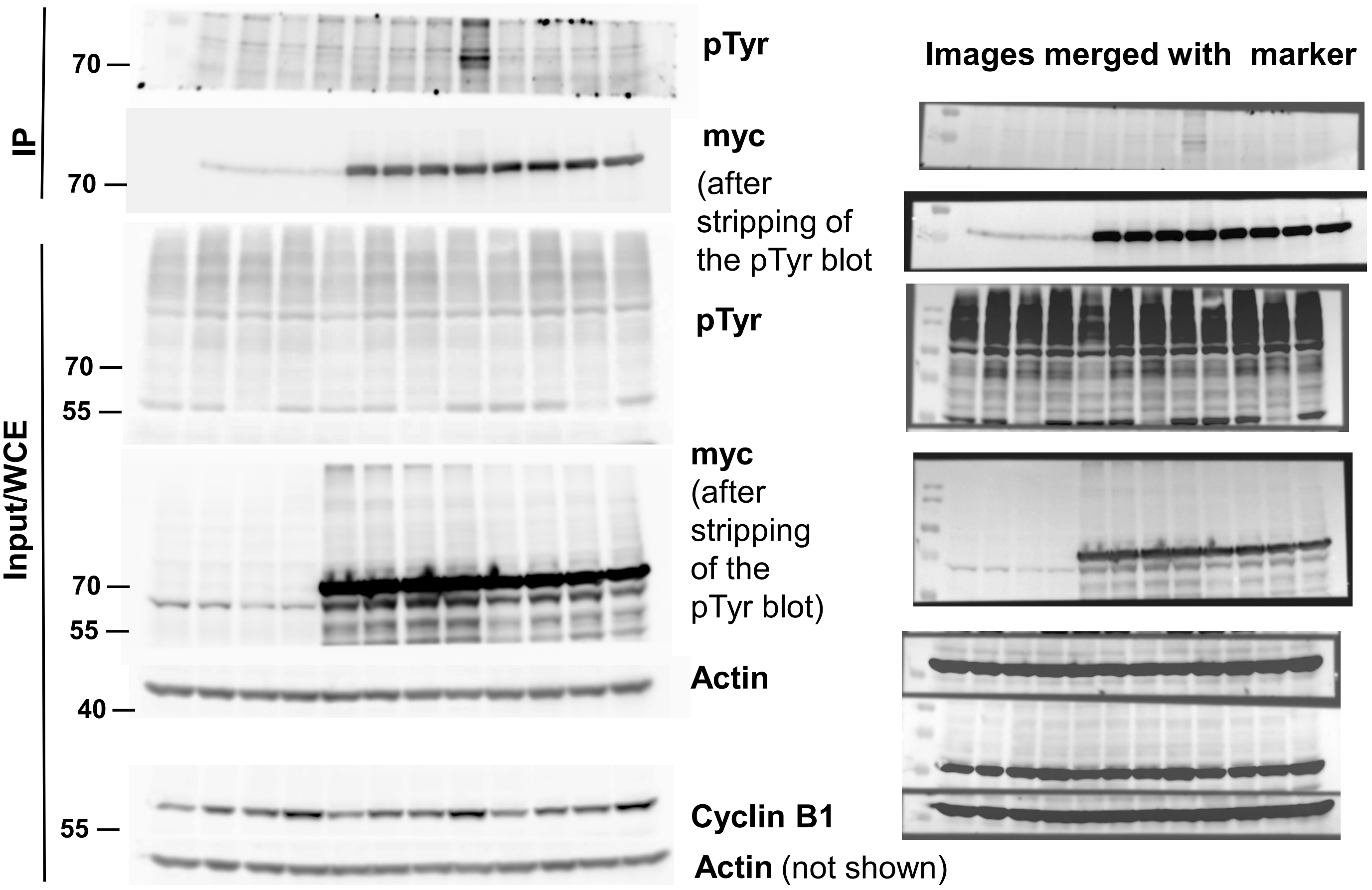

Figure 1 D

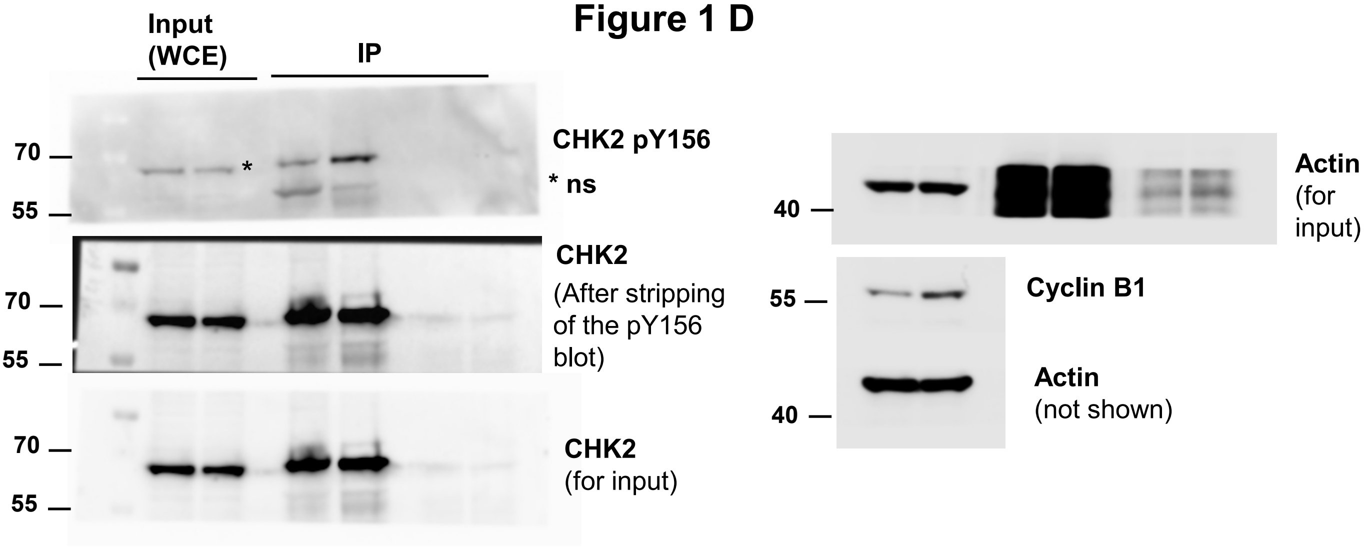

Figure 2 A

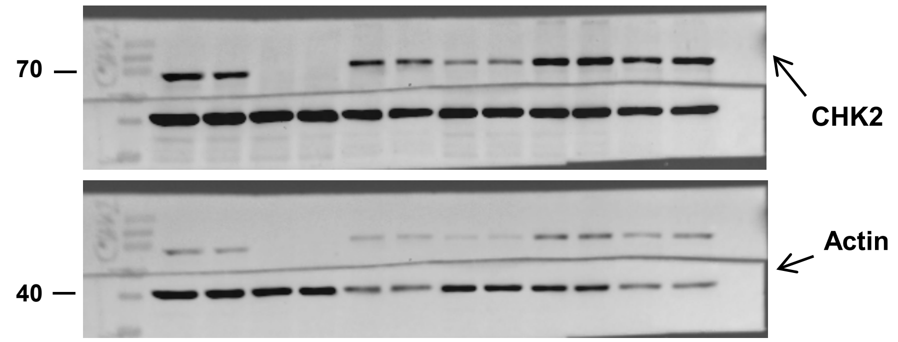

(two different exposure)

Figure 3 A

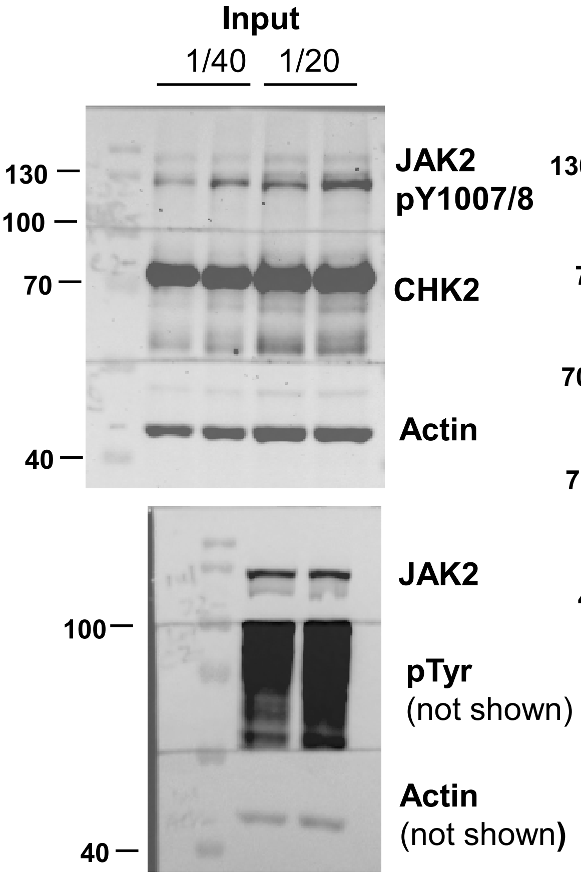

Figure 3 B

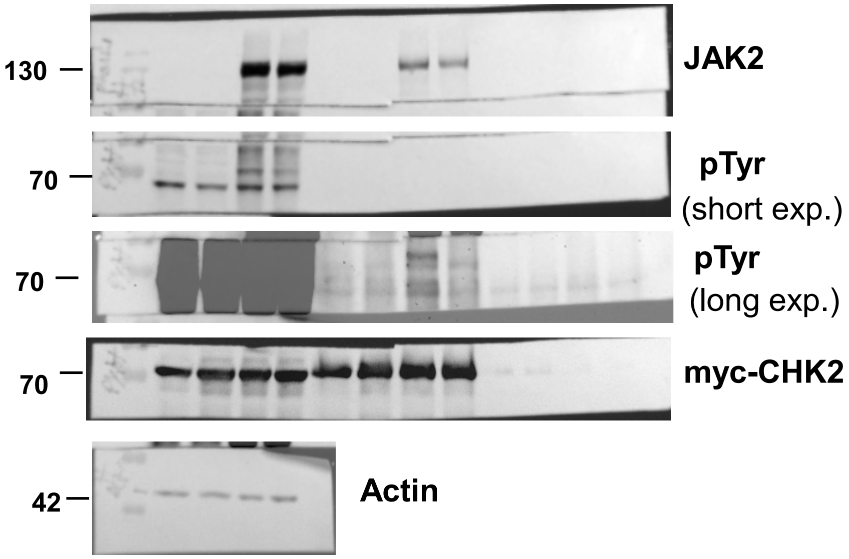

Figure 3 C

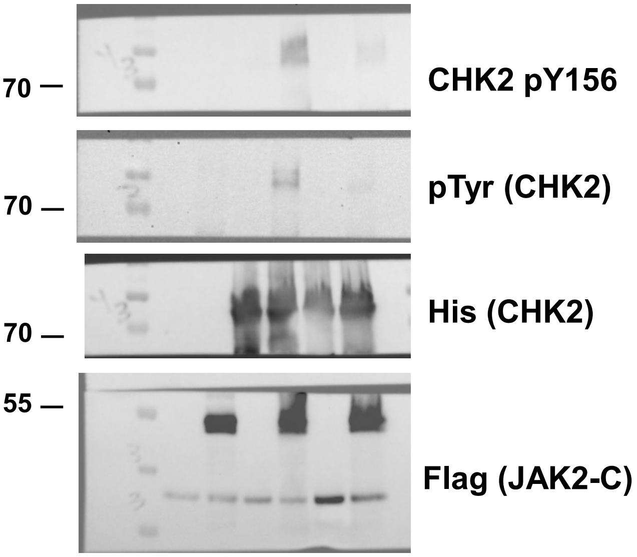

Figure 3 D

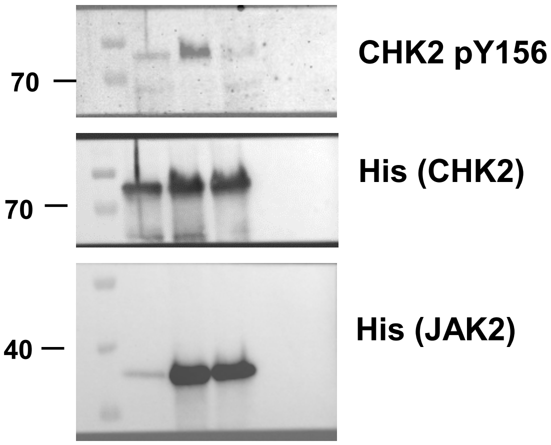

Figure 3 E

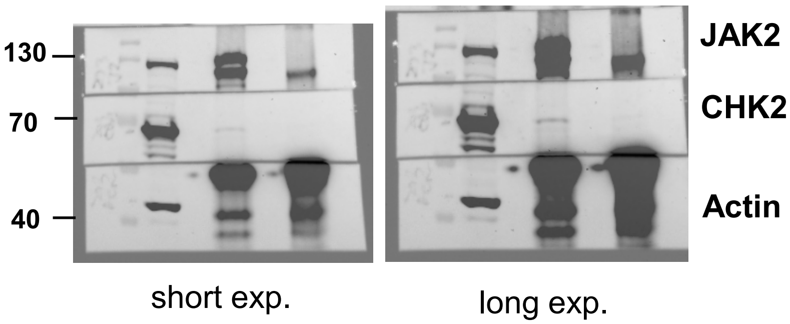

Figure 3 F

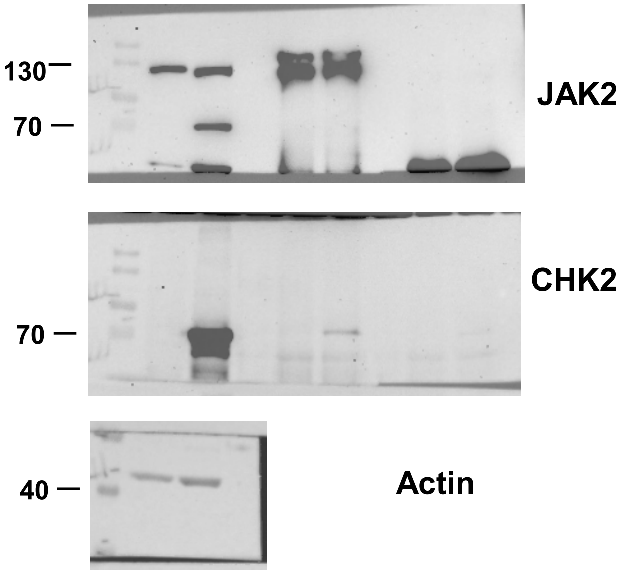

Figure 3 G

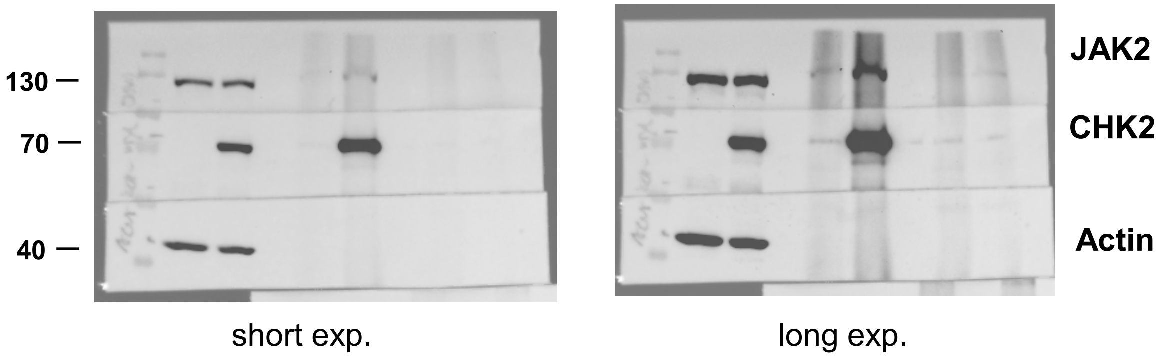

Figure 3 H

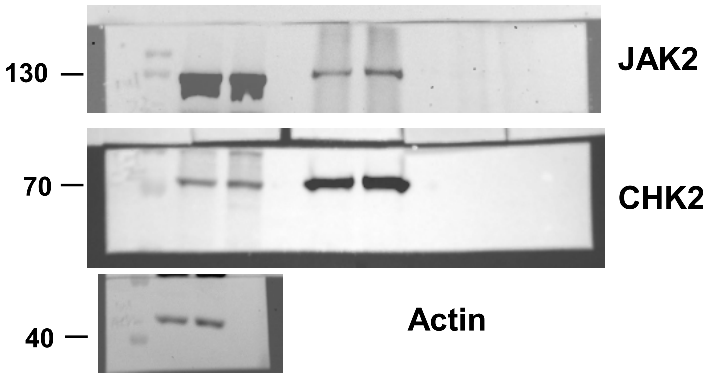

Figure 3 L

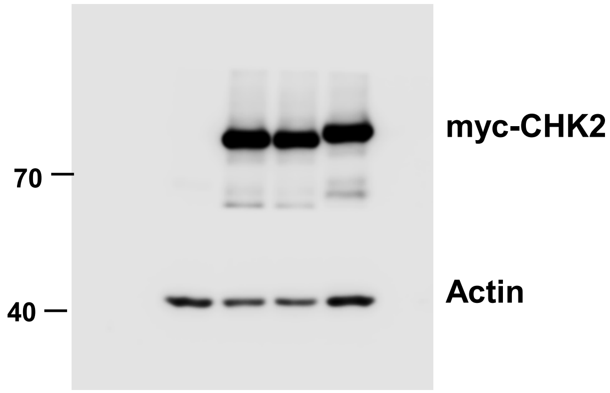

Figure 5 A

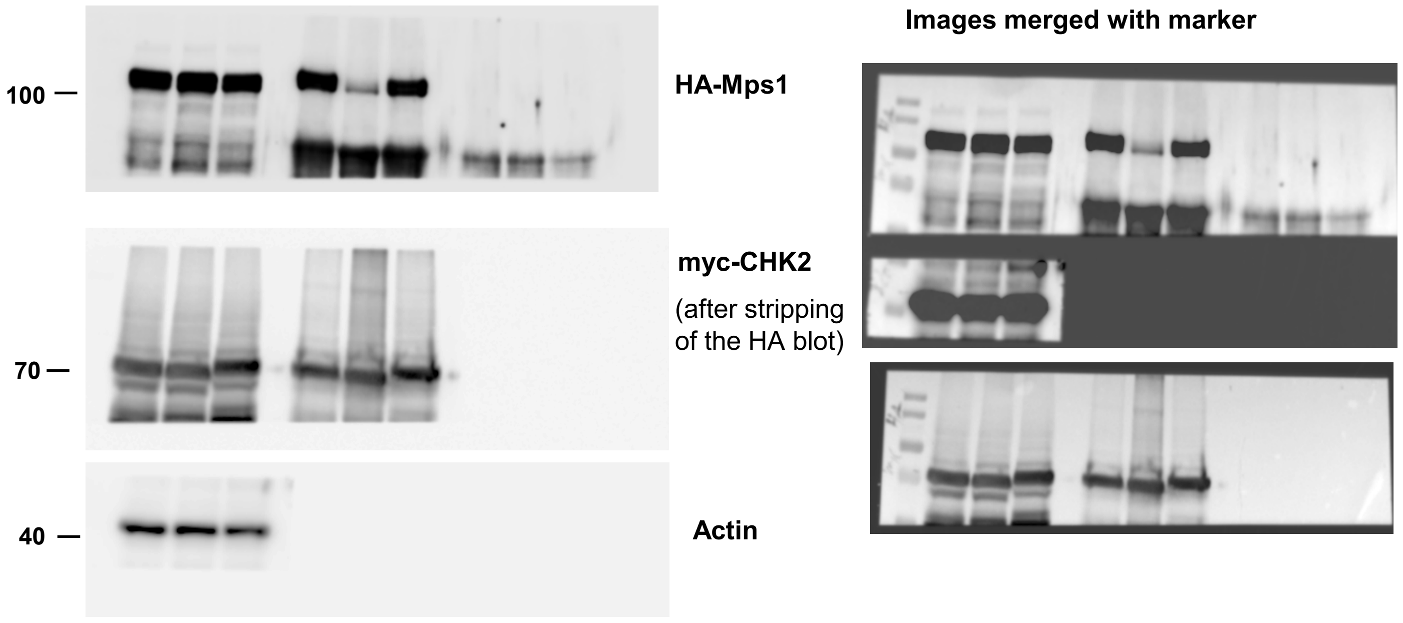

Figure 5 B

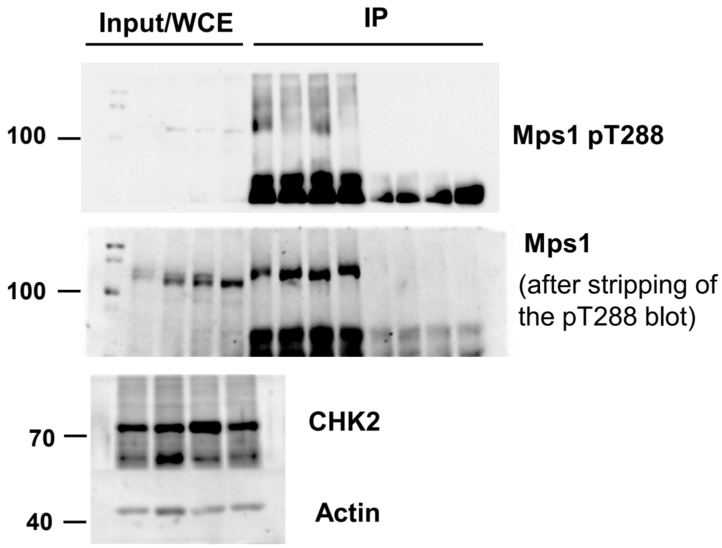

Figure 5 G

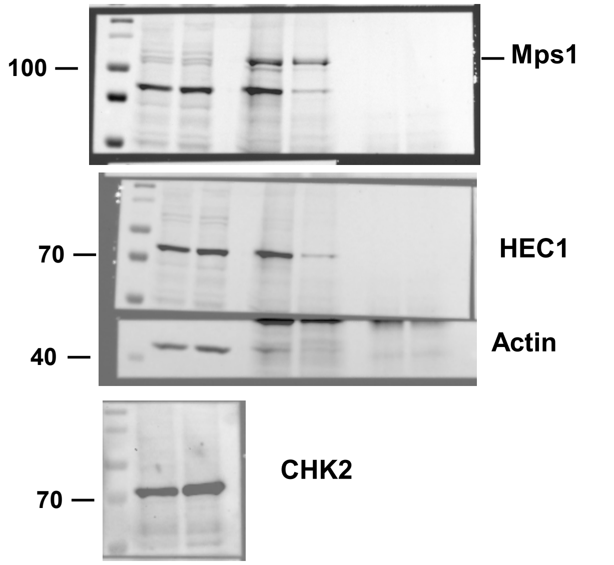

Figure 5 C

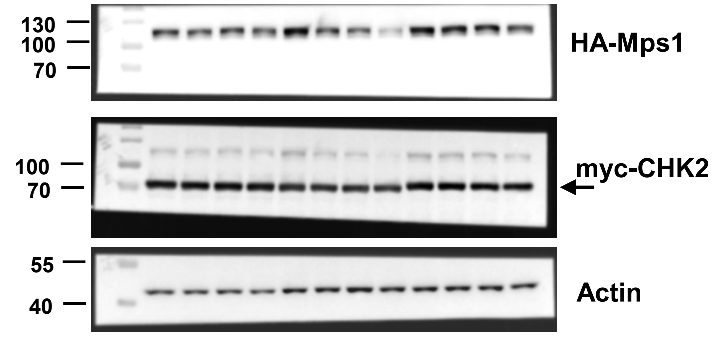

Figure 6 F

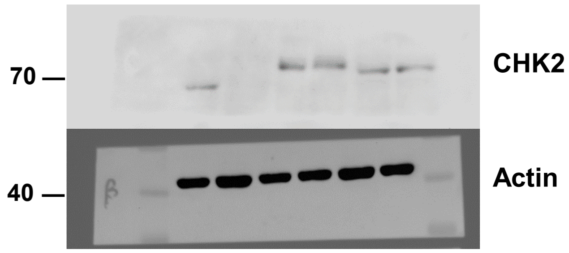

Figure S2A

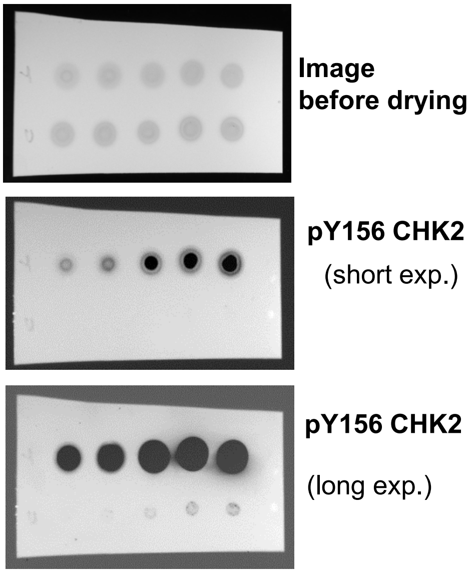

Figure S2B

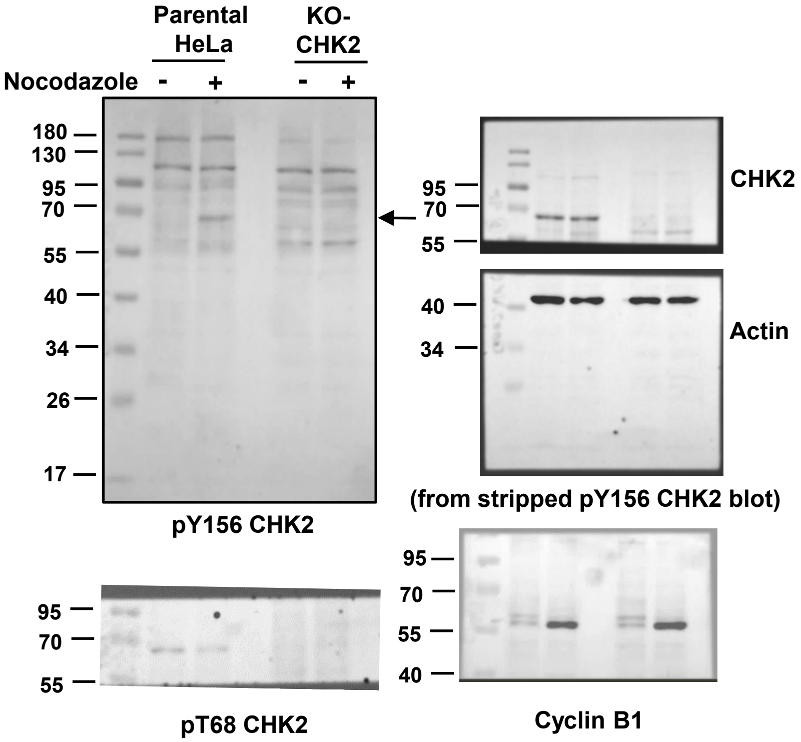

Figure S2C

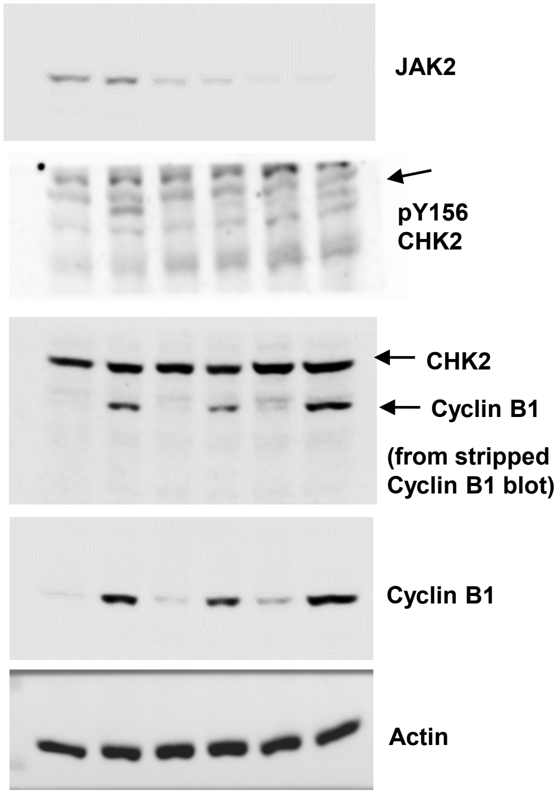

Figure S2D

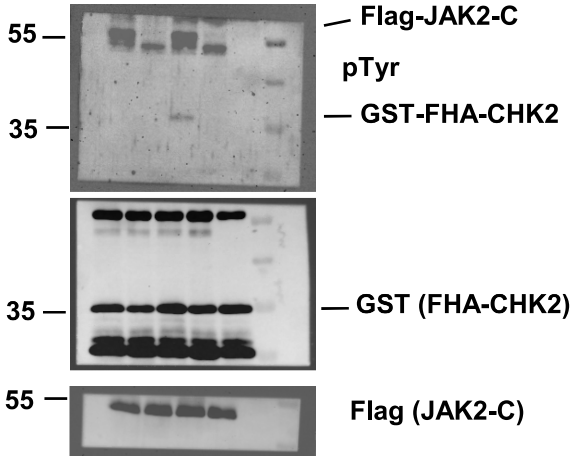

Figure S2E

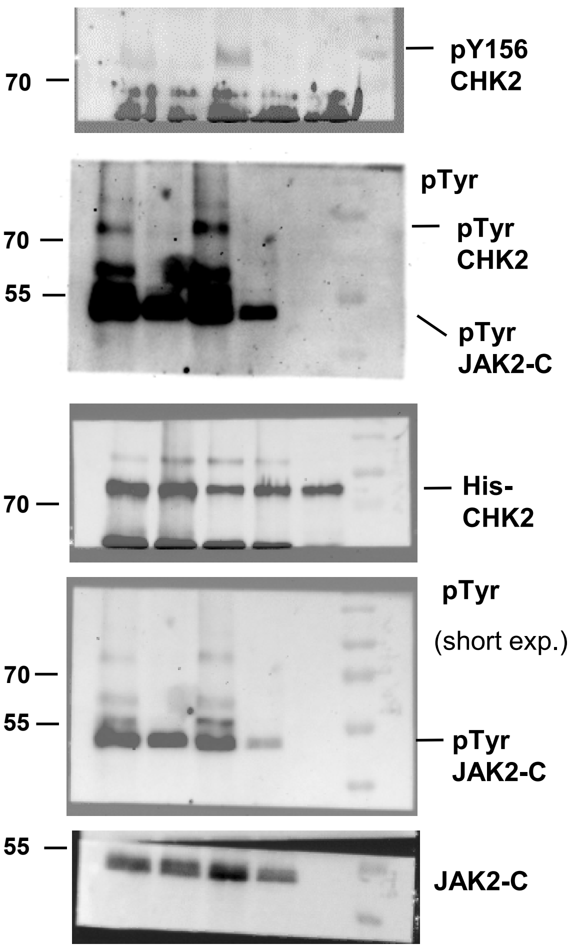

Figure S2F

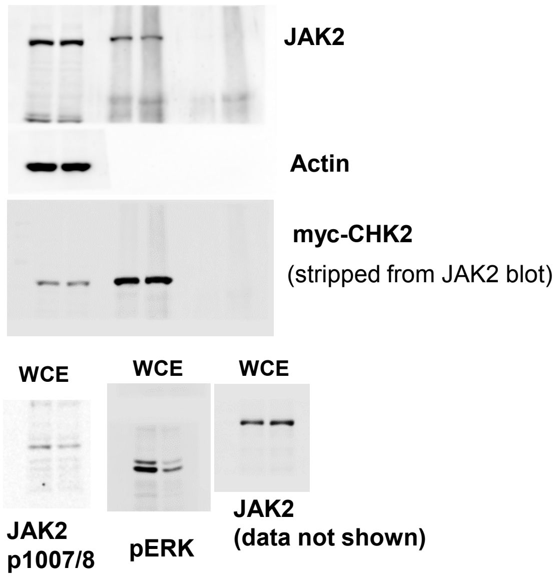

Figure S5A

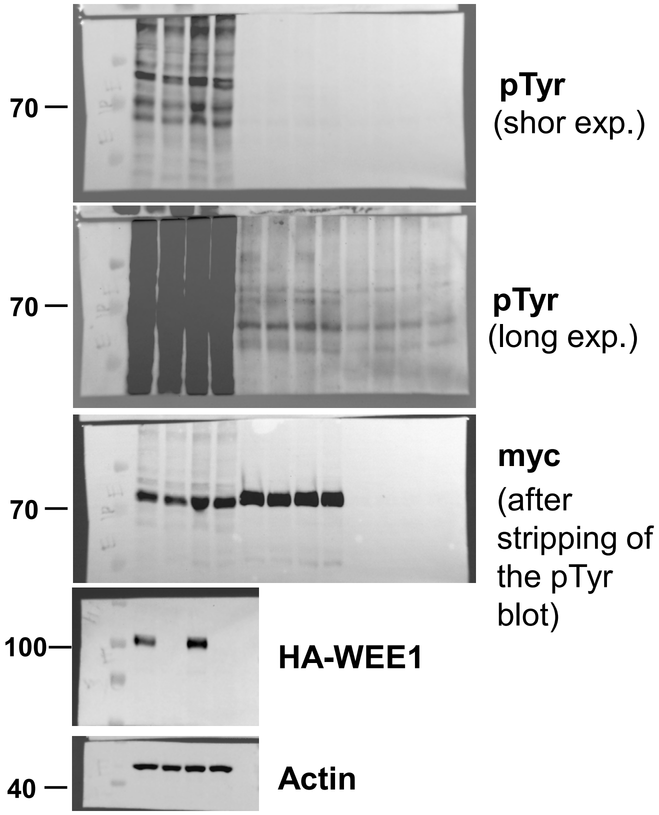

Figure S5B

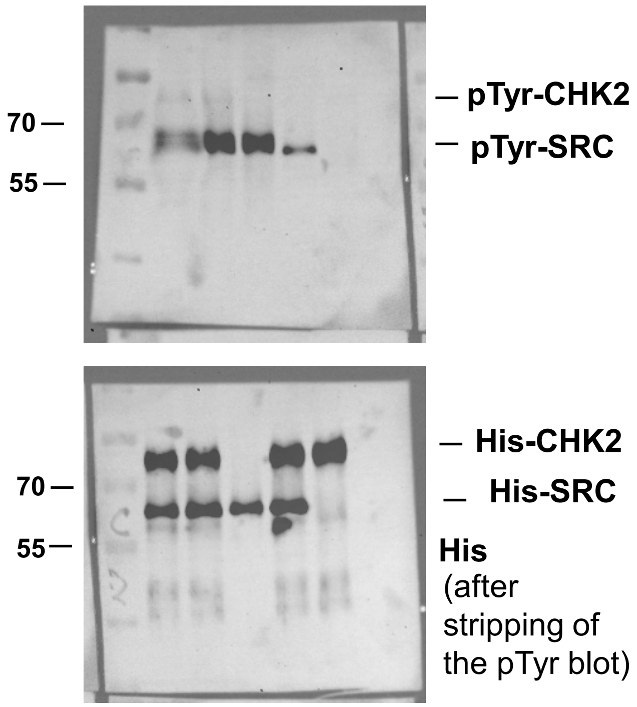

Figure S5 C

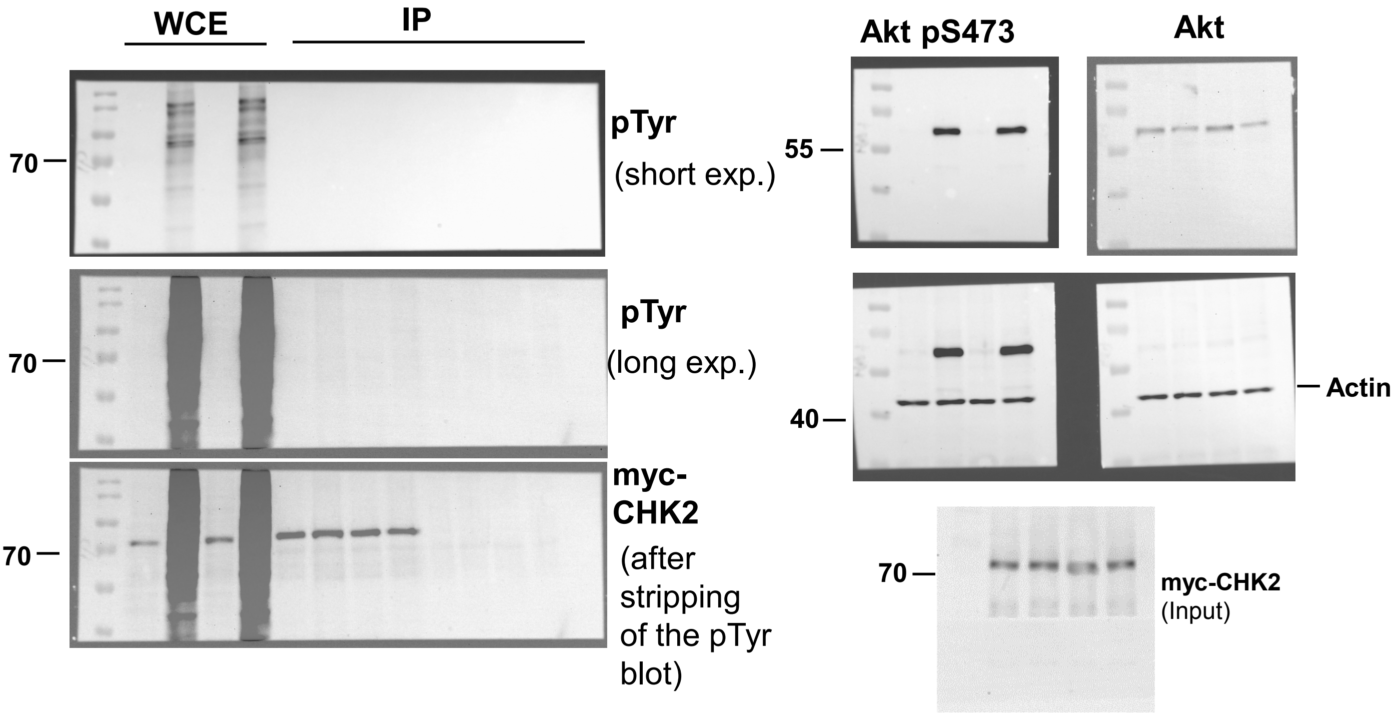

Figure S6C

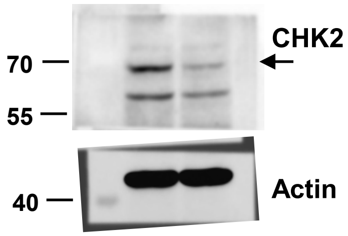

Figure S7A

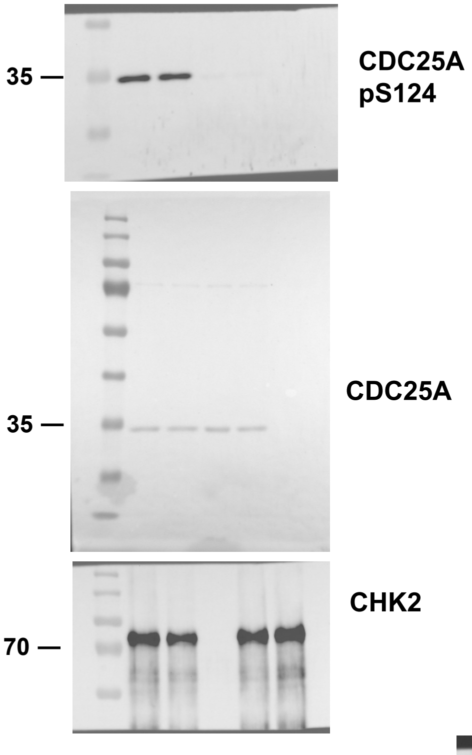

Figure S7B

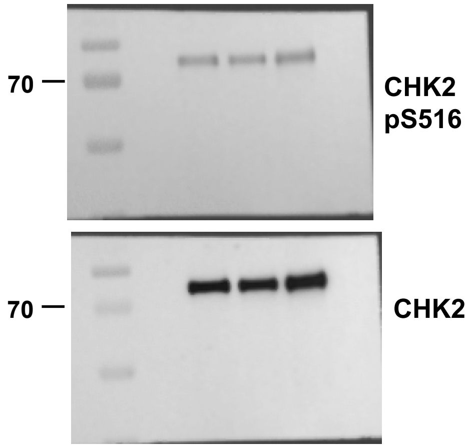

Figure S7C

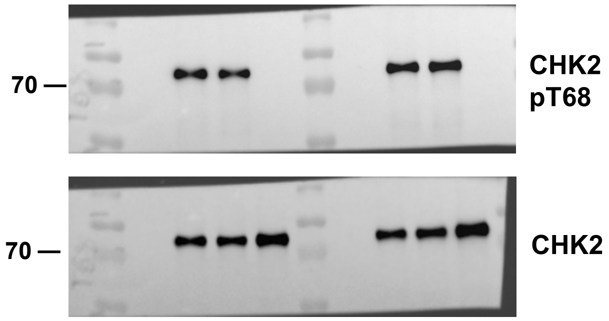

(two sets with different amount loaded)

Figure S8A

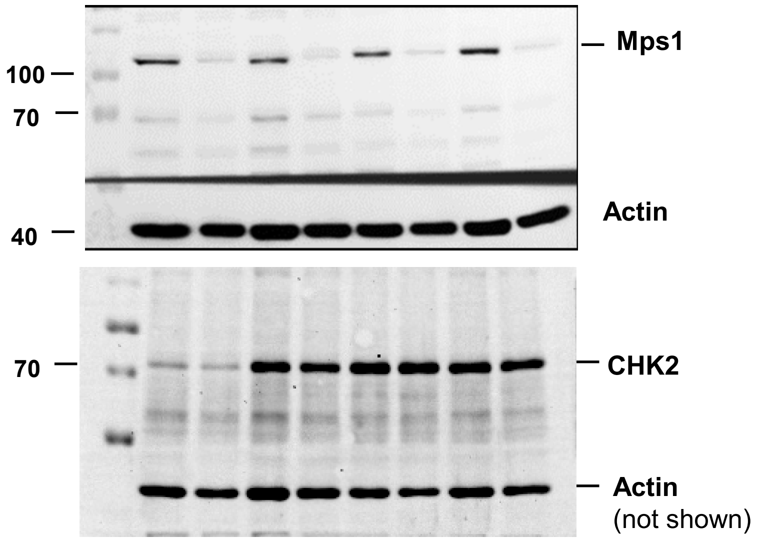

Figure S9A

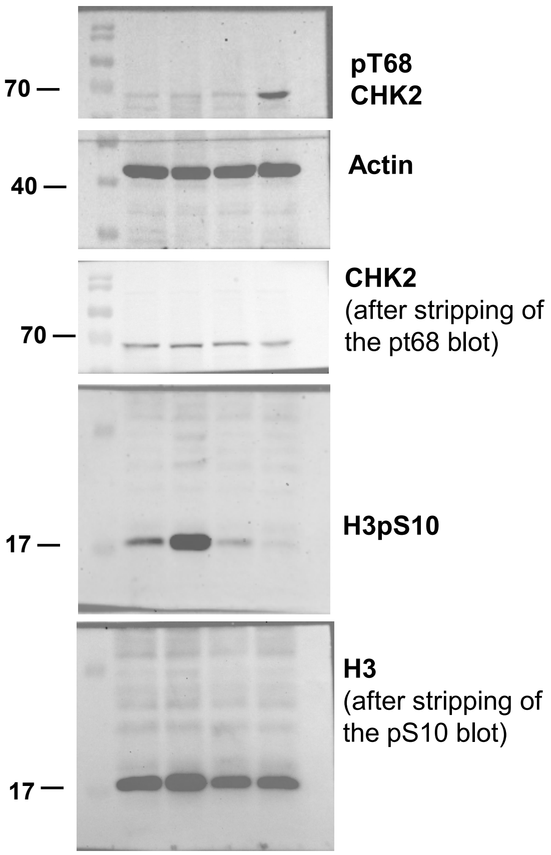

Figure S9B

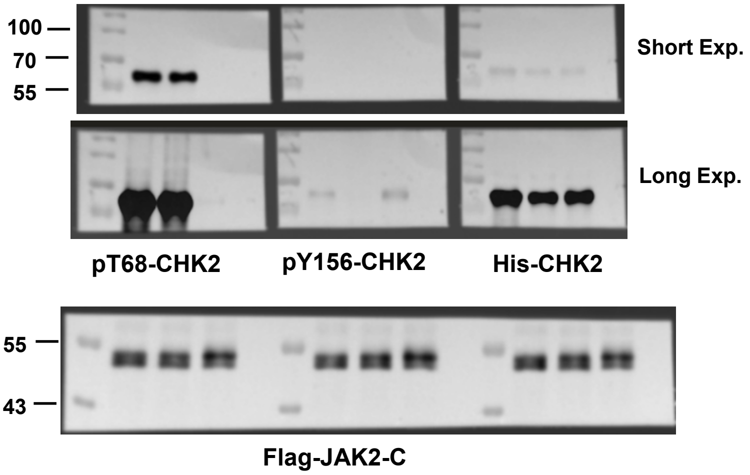

Figure S9C

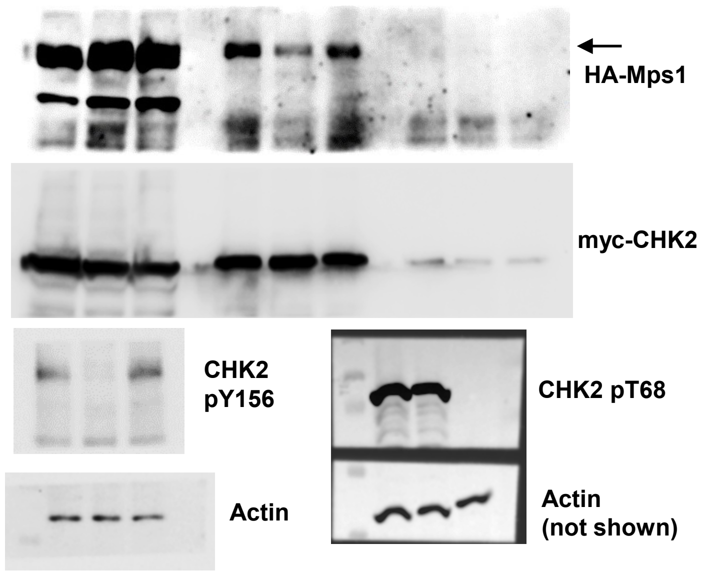

Figure S9D

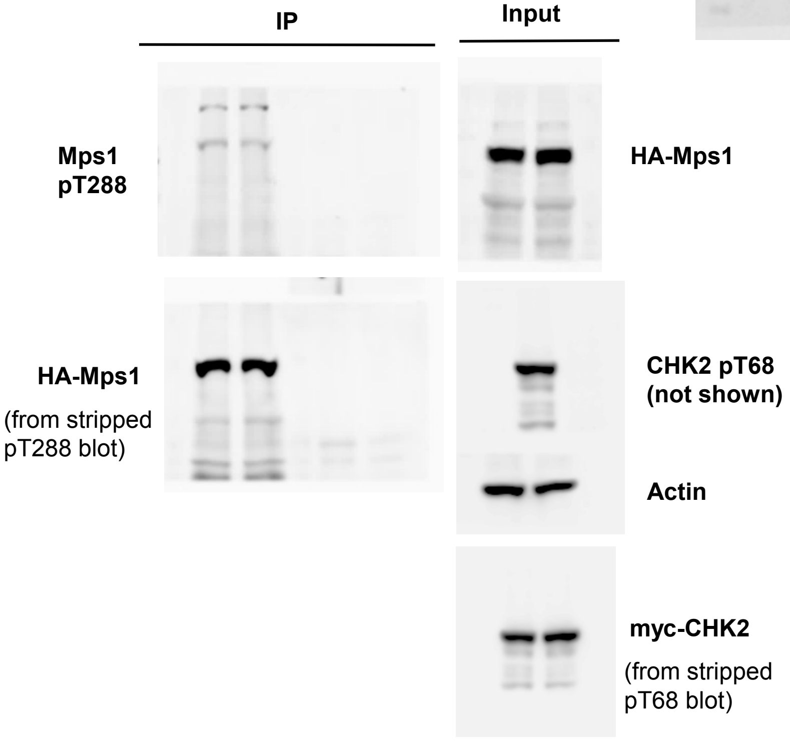

Figure S9E

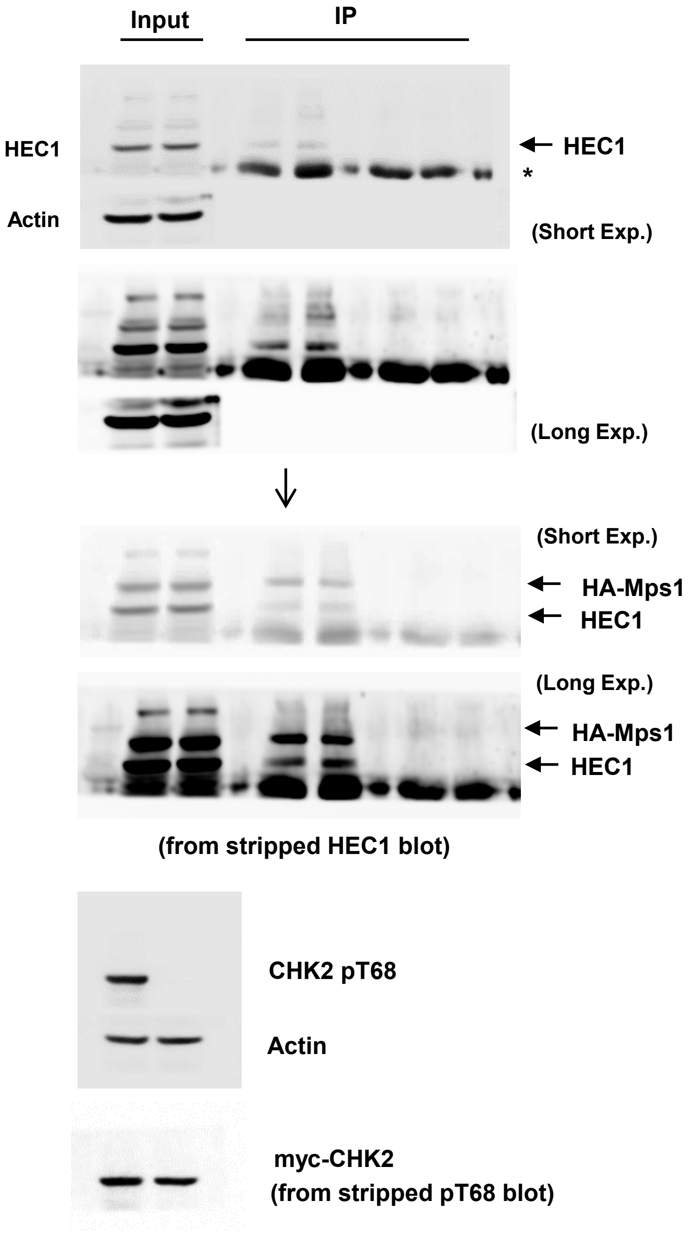

Figure S9F

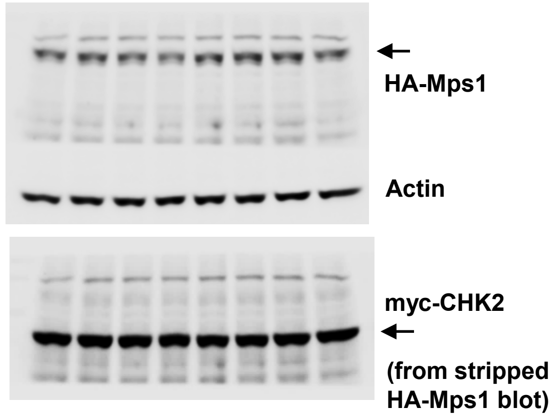

Supplement: Supplementary file 6 — uncropped images [file 41419_2022_5077_MOESM6_ESM.pdf]
